# Perinatal factors influencing the earliest establishment of the infant microbiome

**DOI:** 10.20517/mrr.2024.92

**Published:** 2025-06-12

**Authors:** Kevin Linehan, Kiera Healy, Eimear Hurley, Carol Anne O’Shea, C. Anthony Ryan, R. Paul Ross, Catherine Stanton, Eugene M. Dempsey

**Affiliations:** ^1^Teagasc Food Research Centre, Fermoy, Cork P61 C996, Ireland.; ^2^APC Microbiome Ireland, Biosciences Institute, University College Cork, Cork T12 YT20, Ireland.; ^3^School of Microbiology, University College Cork, Cork T12 YN60, Ireland.; ^4^Department of Paediatrics & Child Health and INFANT Centre, University College Cork, Cork T12 YN60, Ireland.; ^5^VISTAMILK RESEARCH Centre, Teagasc Moorepark, Cork P61 C996, Ireland.

**Keywords:** Infant microbiome, vertical microbial transmission, maternal microbiota, perinatal factors, meconium

## Abstract

**Background:** While extensive research exists on the human microbiome, a number of outstanding questions remain regarding the infant microbiome in the initial stages of life. This study aimed to determine the timing of very early microbial colonization in humans, assess the contribution of maternal microbial sources to their offspring and examine the effects of perinatal factors such as delivery mode, gestational age, and feeding practices on the maternal and infant microbiota in early life.

**Methods:** Using a cohort of 18 healthy mother-infant dyads, maternal saliva (within 24 h postpartum), vaginal (1 h prepartum), and placental (1 h postpartum) samples were collected. From their corresponding infants, saliva (within 24 h postpartum) and meconium (within 96 h postpartum) samples were collected. 16S rRNA amplicon sequencing was utilized to assess the taxonomic and inferred functional compositions of the bacterial communities from both mothers and infants.

**Results:** Our results consolidate and corroborate recent findings addressing the existence of a meconium microbiome and the absence of a placental microbiome. We show that significant sharing of microbiota, primarily *Streptococcus* and *Veillonella* species, between the maternal oral cavity and the infant oral cavity occurs in early life. Perinatal factors such as vaginal delivery and exclusive breastfeeding were strongly associated with enhanced microbial richness and diversity in infants.

**Conclusions:** This study provides information on the relationship between health and delivery factors and the first establishment of the infant microbiota. These findings could offer valuable guidance to clinicians and mothers in optimizing the infant microbiota toward health during infancy and later life.

## INTRODUCTION

The human microbiome comprises diverse microbial communities across various body sites, including the skin, respiratory tract, and gastrointestinal system, significantly influencing host health and disease^[1]^. The microbiome acts as a companion from birth to death, and its establishment plays a pivotal role in the development of the immune and nervous systems^[2,3]^. Aberrant development of the infant gut microbiota is associated with an increased risk of colonization by pathogens, impaired growth, and diseases such as allergies, asthma, and inflammatory bowel disease^[4,5]^. Thus, the formation and development of the infant gut microbiome are critical for ensuring long-term health.

A crucial topic of study pertaining to the infant microbiome is the exact timing of microbial colonization. At present, the initial microbial colonization of humans remains controversial^[6]^. Some studies suggest the presence of microorganisms in prenatal environments, such as the placenta and amniotic fluid, while others attribute such findings to contamination in low-biomass samples^[6,7]^. Neonates born at term are not immunologically naive and are specifically adapted to cope with abrupt exposure to microbial, dietary, and environmental stimuli^[8,9]^. Understanding the onset of colonization is critical for unraveling immune priming mechanisms and optimizing clinical interventions.

The initial assembly of the infant microbiome is primarily driven by the vertical transfer of microbes from mother to infant^[10-12]^. Maternal sources, including the gut, skin, oral cavity, vagina, and breast milk, contribute to this microbial inheritance^[13,14]^. The establishment of the infant microbiota is also influenced by perinatal factors such as delivery mode, gestational age, feeding type, maternal health, and antibiotic exposure^[15]^. For example, vaginally delivered infants acquire microbiota from the mother’s birth canal and intestine, while cesarean-section (CS) delivery disrupts this transmission, often resulting in reduced beneficial microbes like *Bifidobacterium* and *Bacteroides* and an increased abundance of opportunistic pathogens^[16]^. Infants born vaginally and exclusively breastfed, without antibiotic exposure, are considered to have the optimal microbial colonization^[17]^.

Despite advances in our understanding, gaps remain in pinpointing the timing of colonization, the maternal sources of microbial transmission, and the impact of perinatal factors on the infant microbiota^[14]^. This study aimed to address three key questions: (1) Is the intrauterine environment sterile, or does microbial colonization begin in utero? (2) What is the contribution of various maternal microbial sources to the infant microbiota? (3) How do perinatal factors drive microbial inheritance and selection in early life? To answer these questions, we focussed on three potential maternal sources of microbial transmission (oral, vaginal, and placental) to the microbiota of their new-born infant (oral and meconium microbiota) and sought to investigate the contribution of numerous transmission routes and the impact of numerous perinatal factors on the initial establishment of the infant gut and oral microbiome.

## METHODS

### Study design, ethics and recruitment

This study was conducted at Cork University Maternity Hospital (CUMH), recruiting 63 healthy mother-infant dyads born full term either naturally or via CS between November 2015 and February 2019 as part of the Oral Placenta Infant Microbiota Study (OPIuM Study). Ethical approval was obtained from the Cork Teaching Hospitals Clinical Research Ethics Committee [ECM 4 (v) 12/08/14]. Infants born < 35 weeks gestation, admitted to the neonatal intensive care unit (NICU), or born to ill mothers were excluded. A subset of 18 dyads with available samples from all sites (vaginal, maternal saliva, placenta, infant saliva, and meconium) was selected for this analysis. Table 1 summarizes the characteristics of the 18 mother-infant dyads. The infants were 56% male and 44% female, with 44% born via CS and 56% vaginally. The mean birth weight was 3,667.2 g (SD ± 386.6), gestational age 39 weeks (SD ± 0.97), and Apgar score 9 (SD ± 1.15). Premature rupture of membranes (PROM) occurred in 39% of births. Feeding included 56% breastfed, 33% formula-fed, and 11.1% unspecified. None of the infants received antibiotics. Among mothers, 56% received antibiotics (two specified as Amoxicillin and Flucloxacillin), and 44% did not. The mean gravida was 3 (SD ± 1.5), and parity was 2 (SD ± 1.3).

**Table 1:** Clinical characteristics of the 18 mother-infant dyads in this study

**Variables**	
**Infant characteristics**	**Cohort (*n* = 18)**
Gender	
Male	10/18 (55.6%)
Female	8/18 (44.6%)
Delivery mode	
Caesarean	8/18 (44.4%)
Vaginal	10/18 (55.6%)
Birth weight (g), mean (SD)	3667.2 (386.6)
Gestational age (in weeks), mean (SD)	39 (0.97)
AGPAR, mean (SD)	9 (1.15)
PROM	
Yes	7/18 (38.9%)
No	11/18 (61.1%)
Feed type	
Breast fed	10/18 (55.6%)
Formula	6/18 (33.3%)
Not noted	2/18 (11.1%)
**Maternal characteristics**	**Cohort (*n* = 18)**
Intrapartum antibiotic	
Yes	10/18 (55.6%)
No	8/18 (44.4%)
Amoxicillin (Augmentin)	1/18 (5.6%)
Flucloxacillin	1/18 (5.6%)
Type of antibiotic not noted	16/18 (88.8%)
Gravida, mean (SD)	3 (1.5)
Parity, mean (SD)	2 (1.3)

Continuous variables are presented as mean (+SD). Categorical variables are presented as number of participants (percentages). SD: Standard deviation; APGAR: appearance, pulse, grimace, activity, and respiration; PROM: premature rupture of membranes.

### Sample collection

Vaginal and saliva samples were collected using CatchAll^TM^ swabs (Cambio, UK)^[18]^. Mid-vaginal samples were collected by midwives or gynecologists within 1 h before delivery and immediately placed on dry ice, then stored at -80 °C. Placental samples were collected by obstetricians within 1 h of delivery; four random cross-sectional pieces were excised to encompass both maternal and fetal sides, and then stored similarly. Maternal saliva was pooled from the floor of the mouth within 24 h postpartum, and infant saliva was collected within four days of delivery. Meconium samples were collected within four days and confirmed to have typical characteristics (dark, sticky, tar-like). Hospital-collected meconium and saliva samples were immediately stored on dry ice at -80 °C. Home-collected samples were stored at 4 °C until retrieved by the research nurse and transported in a temperature-controlled case to the laboratory for long-term storage at -80 °C.

### DNA extractions

DNA extraction for swab samples (vagina and saliva) was performed using the MO BIO PowerSoil DNA Isolation kit (Qiagen, Hilden, Germany), adapting the protocol to process swabs as previously described^[18,19]^. Thawed swabs were cut above the swab head, which was inserted into PowerBead Pro tubes containing 600 μL of solution C1. Samples were homogenized for 3 min at maximum speed using a Mini Beadbeater (BioSpec), followed by incubation at 65 °C for 10 min. DNA was eluted in 50 μL solution C6 as per the manufacturer’s instructions. Meconium samples underwent DNA extraction using a bead-beating and column-based protocol^[20]^, combined with the QIAamp Fast DNA Stool Mini kit (Qiagen, UK)^[21]^. A lysis buffer (500 mM NaCl, 50 mM Tris-HCl pH 8.0, 50 mM EDTA, 4% w/v SDS) was added to bead-beating tubes containing 0.25 g of meconium, followed by homogenization (3 min, maximum speed), incubation at 70 °C for 15 min, and centrifugation (4 °C, 16,000 × *g*, 5 min). Supernatant pooling, ammonium acetate treatment, ethanol washing, and RNAse/proteinase K treatments were performed before DNA elution in 50 μL buffer AE. Placental DNA was extracted using the DNeasy Blood and Tissue Kit (Qiagen)^[22]^. Approximately 0.25 g of frozen tissue was diced with sterile scalpels and suspended in microcentrifuge tubes with tissue lysis buffer (180 μL ALT) and a stainless steel bead. Tissue homogenization was conducted using a TissueLyser II (Qiagen) at 30 Hz for 20 s. After adding proteinase K (20 μL, 20 mg/mL), samples were incubated at 56 °C with intermittent vortexing. DNA was eluted in 50 μL buffer AE. Extraction controls containing 250 μL sterile water were processed alongside samples. DNA concentrations were measured using a Qubit^TM^ 4 Fluorometer (Thermo Fisher Scientific) with a detection limit of 10 pg/μL. All extracted DNA was stored at -30 °C.

### 16S rRNA gene amplification and MiSeq sequencing

The V3-V4 hypervariable region of the 16S rRNA gene was amplified from 90 DNA samples following the Illumina 16S Metagenomic Sequencing Library Protocol. Negative extraction blanks and no-template controls were included throughout. PCR amplification utilized V3-V4 specific primers [Supplementary Table 1]. Reactions included 5 ng/μL template DNA, primers (5 μM), 12.5 μL KAPA2G Robust HotStart ReadyMix and PCR-grade water. PCR conditions were as follows: 95 °C initial denaturation (3 min), 30 cycles (95 °C, 30 s; 55 °C, 30 s; 72 °C, 30 s), followed by 72 °C (5 min), and a 4 °C hold. Amplicons were verified by gel electrophoresis and cleaned with AMPure XP magnetic beads. Illumina sequencing adapters and dual-index barcodes were added in a second PCR. Samples were pooled equimolarly and verified with a Bioanalyzer before sequencing on an Illumina MiSeq (2 × 300 cycle V3 kit).

### Bioinformatics analysis

Raw sequences were assessed using MultiQC^[23]^ and trimmed with Cutadapt (v2.10). Quality filtering, error correction, denoising, merging of paired reads, and chimera removal were conducted using DADA2 (v1.14)^[24]^. Taxonomic classification of amplicon sequence variants (ASVs) was conducted using the SILVA database (v138)^[25]^, with genus- and species-level matches made via *assignTaxonomy* and *assignSpecies* functions. Phyloseq (v1.24) facilitated the integration of metadata, ASV tables, phylogenetic trees, and taxonomic assignments^[26]^. The decontam package (v1.16.0) was used to identify and remove contaminants associated with low DNA concentration or negative controls^[25,27,28]^. Samples with < 5 k reads and genera present in ≤ 10% of samples were excluded. A phylogenetic tree was built using DECIPHER (v2.16.1) and phangorn (v2.5.5)^[29,30]^. Functional predictions were made with PICRUSt2 (v2.5.1) against the IMG database, and abundant KEGG pathways (Level 3) were identified^[31-33]^.

### Statistical analysis

Analyses were conducted in R (v4.1.2) with visualization via ggplot2 (v3.4.1). Core ASVs were determined by presence in ≥ 50% of samples and abundance > 0.001^[34]^. Annotation was further refined using BLASTn against the NCBI 16S rRNA database^[35]^. Five perinatal factors (maternal antibiotic use, delivery mode, feeding type, infant gender, and PROM) were assessed for their impact on the microbiome. Alpha diversity (Chao1, Shannon, Simpson) was calculated using the iNEXT package^[36]^ and differences analyzed via linear models. Principal component analysis (PCA) was performed on clr-transformed values for beta diversity, with zero imputation using the “const” method^[37]^. Aitchison distances were calculated for beta diversity, and differences were analyzed using PERMANOVA (adonis function, vegan package v2.6.4), with Bonferroni-Holm correction. Significance was set at *P* < 0.05. Differentially abundant taxa were identified using ANCOM-BC^[38]^, while KEGG pathway differences were detected with STAMP (v2.1.3)^[39]^.

## RESULTS

### The placental does not contain a discernable microbiota

Sequencing of placental tissues produced 666,018 reads (mean length 251 bp), with an average of 79,820 reads per sample (SD ± 111,793). Post-quality filtering, dereplication, error modeling, denoising, pair merging, and chimera removal (using DADA2 default parameters), 10 samples contained zero reads and were excluded, leaving 8 samples with an average of 29,774 reads (range 8,630-38,251). Out of 964 ASVs detected across the 10 samples, 835 were identified as contaminants by Decontam, representing 43.7%-67% (median 57%) of ASVs per sample. The remaining 129 ASVs could not be taxonomically resolved to the species level. Filtering steps applied to other sample types, which excluded genera present in ≤ 10% of total samples, resulted in no ASVs for placental samples; this step was thus removed for placental analysis. DNA extraction and amplicon PCR blanks were used for comparison. Actinobacteriota, Firmicutes, and Proteobacteria were the only phyla detected across blanks and placental samples [Supplementary Figure 1A]. Five families (*Bacillaceae*, *Corynebacteriaceae*, *Micrococcaceae*, *Streptococcaceae*, *Xanthobacteraceae*) and six genera (*Afipia*, *Bacillus*, *Corynebacterium*, *Enhydrobacter*, *Micrococcus*, *Streptococcus*) were identified [Supplementary Figure 1B and C].

### Site-specific shared microbial taxa between mother and infant

**Figure 1:**
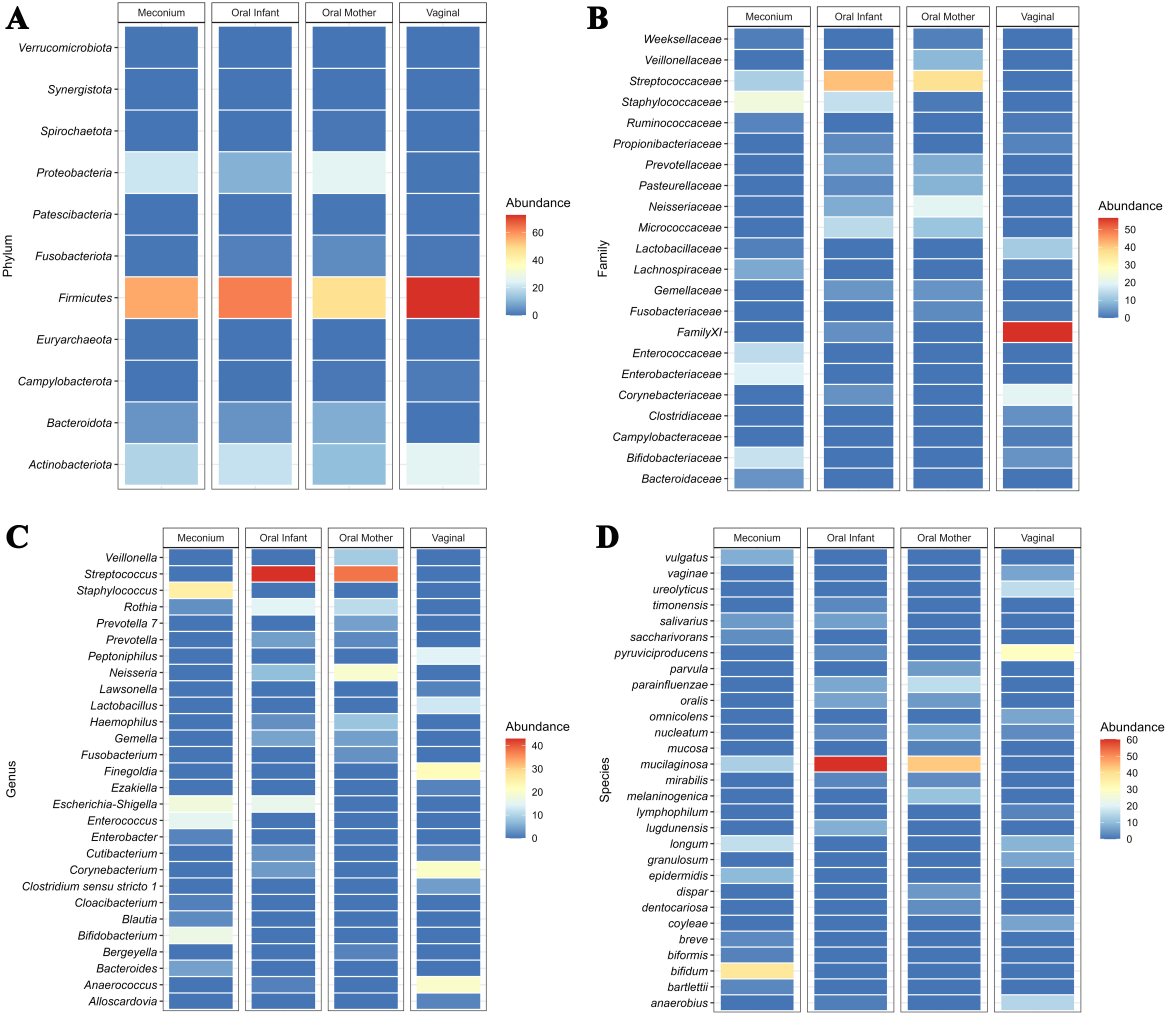
Heat maps of average relative abundances at (A) phylum, (B) family, (C) genus, and (D) species level of meconium, oral saliva, maternal saliva, and vaginal samples.

**Figure 2:**
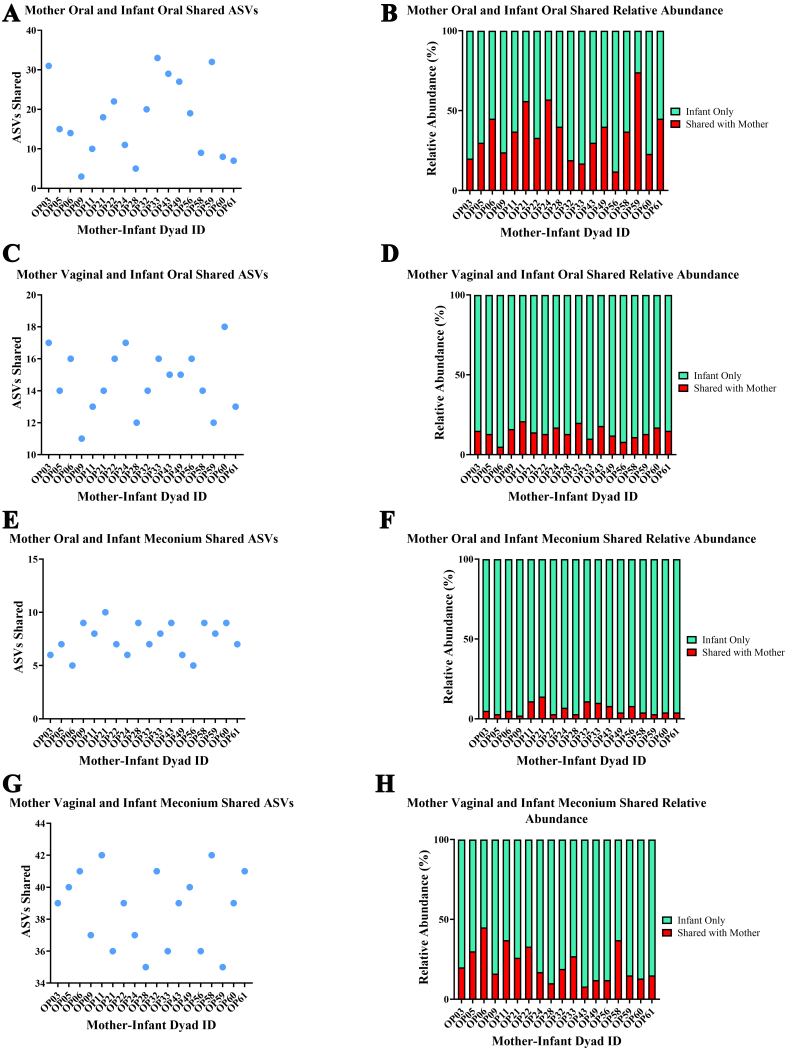
Shared microbiome ASVs between mother and infant samples. (A) Number of ASVs shared between mother oral and infant oral dyad samples; (B) Relative abundance of infant oral microbiome shared with their mother’s oral microbiome; (C) Number of ASVs shared between mother vaginal and infant oral dyad samples; (D) Relative abundance of infant oral microbiome shared with their mother’s vaginal microbiome; (E) Number of ASVs shared between mother oral and infant meconium dyad samples; (F) Relative abundance of infant meconium microbiome shared with their mother’s oral microbiome; (G) Number of ASVs shared between mother vaginal and infant meconium dyad samples; (H) Relative abundance of infant meconium microbiome shared with their mother’s vaginal microbiome. ASVs: Amplicon sequence variants.

An overview of the phylogenetic diversity, core microbiome and metabolic pathways in infant and maternal samples are shown in Supplementary Figures 2-5. To explore site-specific shared bacterial taxa of maternal microbiota species to the infant, we identified bacterial taxa shared between the communities of mothers and their related infants. Figure 1 gives an overview of the overall microbial composition and the relative abundances of the four sample types investigated in this study. Regarding the establishment of the infant’s oral microbiome, on average, a related mother and infant’s oral microbiota shared 45 taxa [Figure 2A], accounting for 65% of the total reads in the infant’s sample [Figure 2B]. Perinatal factors had no effect on the amount of the infants’ oral microbiome that was shared with their mothers. *Rothia mucilaginosa* was found present in all 18 mother-infant oral sample dyads. *Streptococcus oralis*, *Haemophilus parainfluenzae* and *Fusobacterium nucleatum* were found in 15 dyads. On average, a related mother’s vaginal microbiome and the infant’s oral microbiota shared 15 bacterial taxa [Figure 2C], which accounted for 15% of the total reads in the infant’s sample [Figure 2D]. When comparing natural birth (NB) infants with those born by CS, we found that the mode of delivery had a significant effect on the amount of the infants’ oral microbiome that was shared with their mother’s vaginal microbiome (Mann Whitney Test, *P* = 0.045) [Supplementary Figure 6]. *Corynebacterium. pyruviciproducens* was present in all 18 mother-infant dyads when comparing vaginal and oral samples and was the only species common to oral and vaginal samples. *F. nucleatum* was present in 15 dyads. On average, a related mother’s oral microbiota and the infant’s meconium microbiota shared eight bacterial taxa [Figure 2E], which accounted for 6% of the total reads in the infant’s sample [Figure 2F]. We next examined if the amount of the infants’ meconium microbiome that was shared with their mother’s oral microbiota differed according to the various perinatal factors investigated in previous sections; however, no significant correlations were found. Regarding species sharing, *R. mucilaginosa* was the only shared species and was present in all 18 mother-infant dyads. On average, a related mother’s vaginal microbiota and the infant’s meconium microbiota shared 38 bacterial taxa [Figure 2G], which accounted for 21% of the total reads in the infant’s sample [Figure 2H]. We next examined if the amount of taxa in the infants’ meconium that was shared with their mother’s oral microbiome differed according to the various perinatal factors investigated in previous sections; however, no significant correlations were observed. Regarding species sharing, *B. longum* was the only shared species.

### Impact of perinatal factors on the meconium microbiome

**Figure 3:**
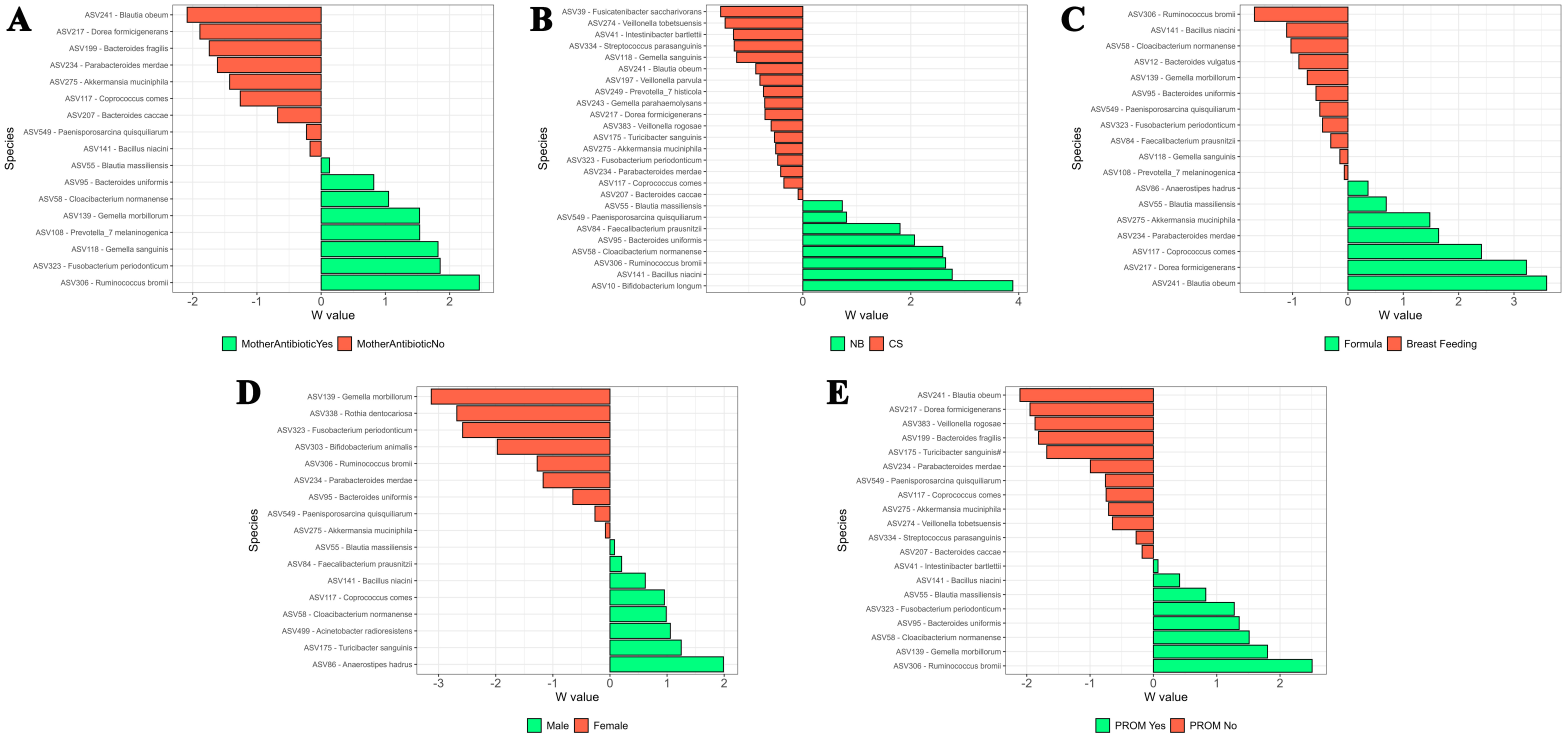
Bar plots of differentially abundant ASVs within meconium samples at the species level. Perinatal factors are shown as green (representing one condition) and red (representing another condition). (A) Mother antibiotic usage; (B) Delivery mode; (C) Feed type; (D) Gender; (E) PROM. Differentially abundant ASVs were identified using the ANCOM method. Only statistically significant ASVs that fall within a distribution based on W values, are shown. ASVs: Amplicon sequence variants; PROM: premature rupture of membranes; ANCOM: analysis of composition of microbiomes.

Maternal antibiotic usage modestly impacted meconium beta diversity (R^2^ = 0.0764, *P* = 0.069). In the “no” antibiotic group, 16 ASVs were differentially abundant at the genus level, including *Eubacterium hallii* group and *Dialister*, with 8 significant ASVs at the species level, such as *Blautia obeum* and *Bacteroides fragilis* [Figure 3A]. In the “yes” group, 15 ASVs, including *Pantoea* and *Enterobacter*, were significant at the genus level, with 8 species-level differences, including *Ruminococcus bromii* and *Gemella sanguinis* [Figure 3A]. The “yes” group was also associated with the enrichment of heme biosynthesis pathways [Supplementary Figure 7A]. C-section delivery increased species richness (Chao1, *P* = 0.07). NB infants had 10 differentially abundant ASVs at the genus level, such as *Cloacibacterium* and *Bifidobacterium*, and 8 at the species level, including *Bifidobacterium longum* [Figure 3B]. CS-born infants showed 21 significant ASVs at the genus level, including *Fusicatenibacter* and *Eubacterium eligens*, with 7 species-level differences like *Veillonella tobetsuensis* [Figure 3B]. NB infants were associated with heterolactic fermentation [Supplementary Figure 7B]. Breastfeeding was associated with 21 differentially abundant ASVs at the genus level, including *Delftia*, and 11 at the species level, such as *Bacteroides vulgatus* [Figure 3C]. Formula-fed infants showed 14 genus-level differences, including *Eubacterium eligens* group, and 7 species-level differences, such as *Blautia obeum* [Figure 3C]. Breastfeeding was associated with fatty acid oxidation pathways [Supplementary Figure 7C]. Gender differences revealed 10 significant ASVs in males, including *Gemella*, and 11 at the species level, such as *Anaerostipes hadrus* [Figure 3D]. Females showed 15 genus-level differences, including *Massilia*, with 7 species-level differences like *Rothia dentocariosa* [Figure 3D]. For PROM births, 29 ASVs were more abundant in the “no” group at the genus level, including *Eubacterium eligens* group, with 12 species-level differences, such as *Blautia obeum* [Figure 3E]. The “yes” group showed 8 genus-level differences, including *Delftia*, and 8 species-level differences, such as *Ruminococcus bromii* [Figure 3E].

### Impact of perinatal factors on the infant oral microbiome

**Figure 4:**
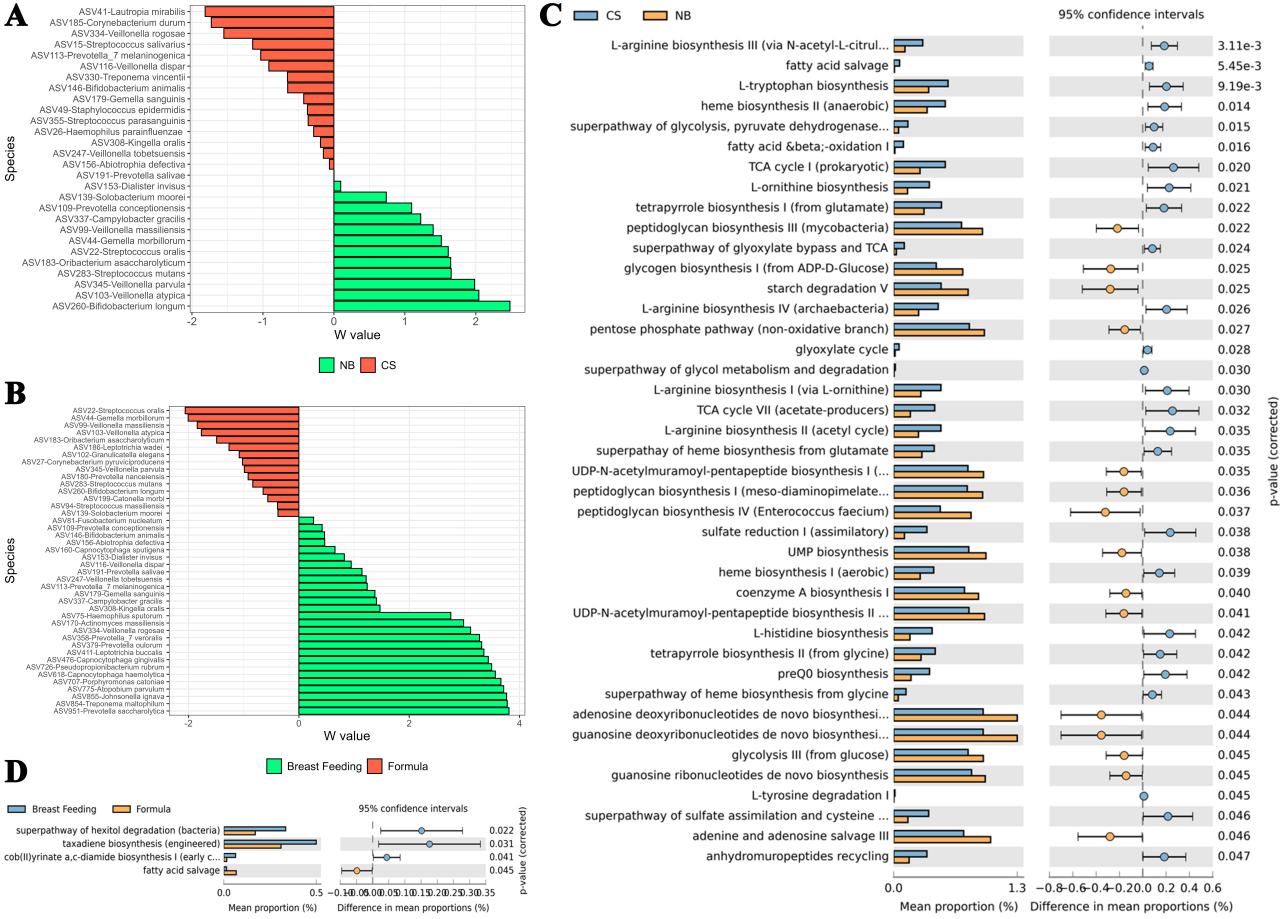
Bar plots of differentially abundant ASVs within infant oral samples at the species level and the impact of perinatal factors on oral microbiome functionality. (A) Delivery mode. (B) Feed type. Differentially abundant ASVs were identified using the ANCOM method, with only statistically significant ASVs based on W values shown (see Supplementary Table 1 for W values); (C) Delivery mode: Functional pathways associated with NB and CS groups; (D) Feed: Functional pathways associated with the “Breast Feeding” and “Formula” groups. (C and D) Extended error bar plots represent the mean proportion of PICRUSt2-predicted KEGG functions, with error bars showing the difference between groups. Bar color indicates the group with the higher proportion for the respective function. Welch’s t-test for unequal variances was applied. Statistical significance was set at *P* < 0.05 (two-sided) following adjusted measures. ASVs: Amplicon sequence variants; ANCOM: analysis of composition of microbiomes; NB: natural birth; CS: cesarean-section.

In this study, maternal antibiotic usage, PROM, and gender had no significant effects on the infant oral microbiome. Regarding delivery mode, six ASVs were differentially abundant in NB infants at the genus level, most significantly *Parvimonas*, *Anaeroglobus*, and *Oribacterium*. At the species level, 12 ASVs were differentially abundant in NB infants, most significantly *Bifidobacterium longum*, *Veillonella atypica*, and *Veillonella parvula* [Figure 4A]. In CS-born infants, 18 ASVs were differentially abundant at the genus level, most significantly *Lautropia*, *Enhydrobacter*, and *Kocuria*, with 16 ASVs differentially abundant at the species level, including *Lautropia mirabilis* (*L*. *mirabilis*), *Corynebacterium durum*, and *Veillonella rogosae* [Figure 4A]. NB infants were associated with peptidoglycan biosynthesis, while CS-born infants were enriched in L-tryptophan biosynthesis and fatty acid salvage pathways [Figure 4B and C]. Feed type had a significant effect on beta diversity (R^2^ = 0.1148, *P* = 0.049). In breastfed infants, 27 ASVs were differentially abundant at the genus level, most significantly the *Eubacterium brachy* group, *Parvimonas*, and *Anaeroglobus*, with 27 species-level ASVs, including *Prevotella saccharolytica*, *Treponema maltophilum*, and *Johnsonella ignava* [Figure 4B]. Formula-fed infants had 14 genus-level differences, most significantly *Lautropia*, *Acinetobacter*, and *Escherichia-Shigella*, with 15 species-level differences, including *Streptococcus oralis*, *Gemella morbillorum*, and *Veillonella atypica* [Figure 4C]. Breastfeeding was associated with hexitol degradation, while formula feeding was linked to fatty acid salvage [Figure 4D].

### Impact of perinatal factors on the maternal oral microbiome

**Figure 5:**
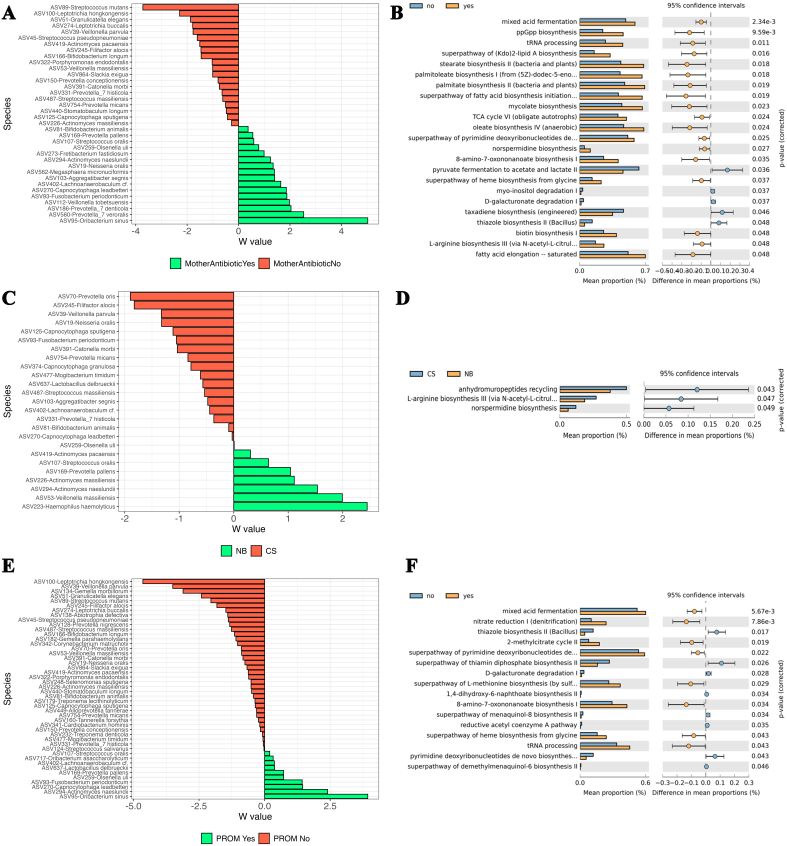
Bar plots of differentially abundant ASVs at the species level and functional pathways in the maternal oral microbiome, based on perinatal factors. (A) Antibiotic usage: Bar plots of significant ASVs at the species level in the “yes” and “no” groups; (B) Delivery mode: Bar plots of significant ASVs at the species level in the NB and CS groups; (C) PROM: Bar plots of significant ASVs at the species level in the “yes” and “no” groups; (D) Antibiotic usage: Functional pathways associated with the “yes” and “no” groups; (E) Delivery mode: Functional pathways associated with NB and CS groups; (F) PROM: Functional pathways associated with the “yes” and “no” groups. Differentially abundant ASVs were identified using the ANCOM method, and functional pathways were predicted using PICRUSt2. Statistical significance was set at *P* < 0.05 (two-sided). ASVs: Amplicon sequence variants; NB: natural birth; CS: cesarean-section; PROM: premature rupture of membranes; ANCOM: analysis of composition of microbiomes.

Maternal antibiotic usage significantly affected the beta diversity of the maternal oral microbiome (R^2^ = 0.0877, *P* = 0.013). In the “no” antibiotic group, seven ASVs were differentially abundant at the genus level, most significantly *Mycoplasma*, *Filifactor*, and *Anaeroglobus*. At the species level, 20 ASVs were differentially abundant, including *Streptococcus mutans*, *Leptotrichia hongkongensis*, and *Granulicatella elegans* [Figure 5A]. In the “yes” group, four ASVs were differentially abundant at the genus level, most significantly *Oribacterium* and *Mogibacterium*. At the species level, 16 ASVs were significant, including *Oribacterium sinus* and *Prevotella_7 denticola* [Figure 5A]. Mixed acid fermentation was associated with antibiotic exposure [Figure 5B]. Regarding delivery mode, four ASVs were differentially abundant in the NB group at the genus level, such as *Anaerococcus* and *Candidatus Saccharimonas*, while seven ASVs were significantly different at the species level, including *Haemophilus haemolyticus* and *Veillonella massiliensis* [Figure 5C]. CS-born mothers exhibited nine genus-level differences, including *Filifactor* and *Mycoplasma*, and 18 species-level differences, such as *Prevotella oris* and *Veillonella parvula* [Figure 5C]. CS delivery was associated with cell wall recycling pathways [Figure 5D]. PROM significantly affected the beta diversity (R^2^ = 0.0874, *P* = 0.022). In the “no” PROM group, 15 ASVs were significant at the genus level, predominantly *Staphylococcus* and *Filifactor*, with 36 ASVs at the species level, such as *L. hongkongensis* and *V. parvula* [Figure 5E]. In the “yes” group, six genus-level ASVs, including *Scardovia* and *Anaerococcus*, were significantly different, with 10 species-level differences, such as *O. sinus* and *Capnocytophaga leadbetteri* [Figure 5E]. Thiazole biosynthesis was associated with the “no” PROM group, while nitrate reduction was linked to the “yes” group [Figure 5F].

### Impact of perinatal factors on the maternal vaginal microbiome

**Figure 6:**
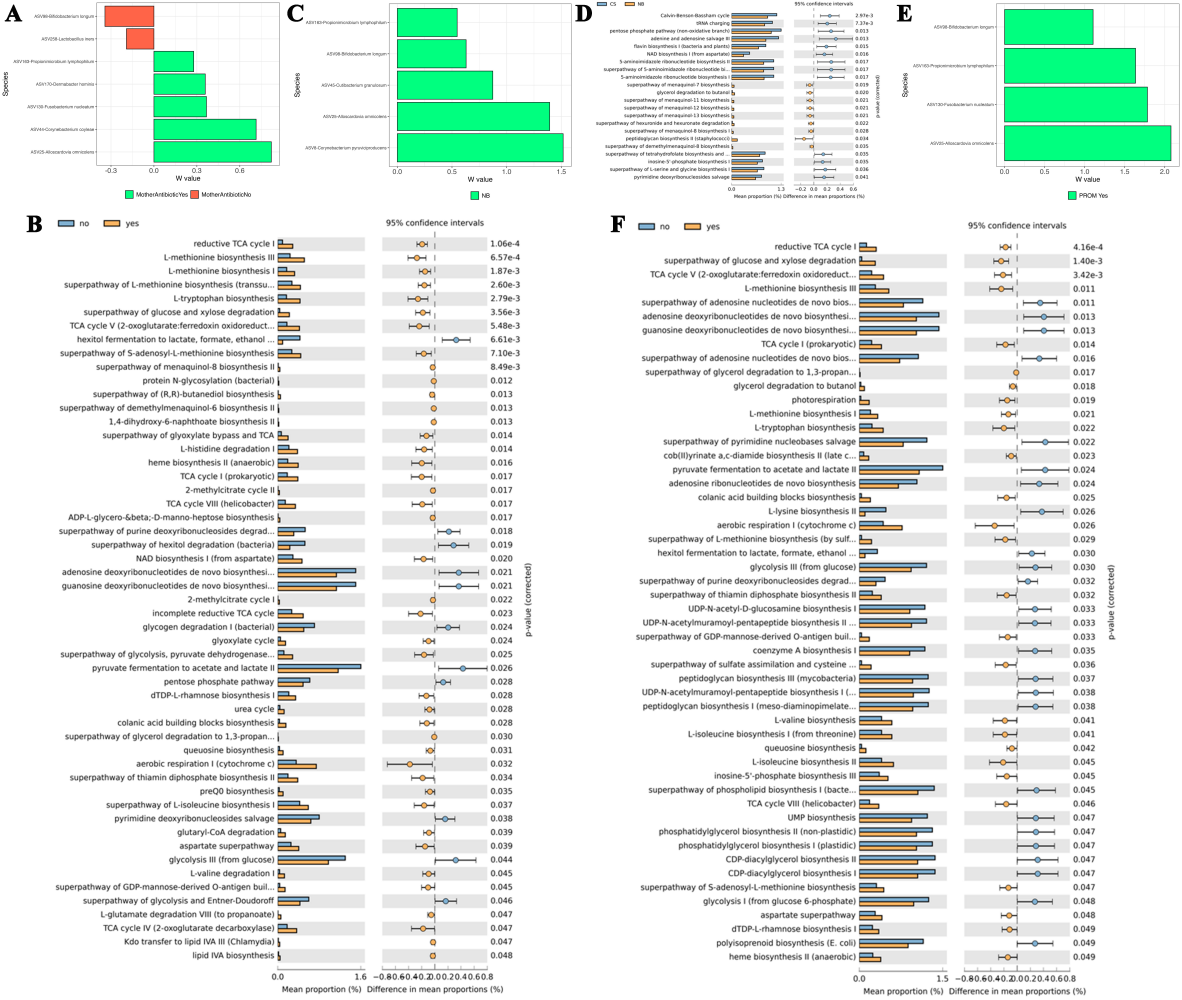
Bar plots of differentially abundant ASVs at the species level and functional pathways in the maternal vaginal microbiome, based on perinatal factors. (A) Antibiotic usage: Bar plots of significant ASVs at the species level in the “yes” and “no” groups; (B) Delivery mode: Bar plots of significant ASVs at the species level in the NB and CS groups; (C) PROM: Bar plots of significant ASVs at the species level in the “yes” and “no” groups; (D) Antibiotic usage: Functional pathways associated with the “yes” and “no” groups; (E) Delivery mode: Functional pathways associated with NB and CS groups; (F) PROM: Functional pathways associated with the “yes” and “no” groups. Differentially abundant ASVs were identified using the ANCOM method, and functional pathways were predicted using PICRUSt2. Statistical significance was set at *P* < 0.05 (two-sided). ASVs: Amplicon sequence variants; NB: natural birth; CS: cesarean-section; PROM: premature rupture of membranes; ANCOM: analysis of composition of microbiomes.

Maternal antibiotic usage significantly influenced the vaginal microbiome. In the “no” group, two ASVs were differentially abundant at the genus level, most notably *Clostridium sensu stricto 1* and *Staphylococcus*. At the species level, *Bifidobacterium longum* and *Lactobacillus iners* were differentially abundant [Figure 6A]. In the “yes” group, nine ASVs were differentially abundant at the genus level, with *Fenollaria*, *Fastidiosipila*, and *Alloscardovia* being the most significant. Five ASVs were differentially abundant at the species level, including *Alloscardovia omnicolens* (*A*. *omnicolens*), *Corynebacterium coyleae*, and *F. nucleatum* [Figure 6A]. Hexitol fermentation was associated with the “no” antibiotic group, while the “yes” group was enriched in pathways including the reductive TCA cycle and L-methionine biosynthesis [Figure 6B]. Regarding delivery mode, CS delivery significantly increased species richness in vaginal samples (Chao1, *P* = 0.091). Following NB, five ASVs were differentially abundant, including *C. pyruviciproducens*, *L. iners*, and *B. longum* [Figure 6C]. NB was associated with pathways such as menaquinol biosynthesis, while CS delivery was linked to the Calvin-Benson-Bassham cycle and pentose phosphate pathway [Figure 6D]. PROM significantly increased alpha diversity (Chao1, *P* = 0.085; Shannon, *P* = 0.015). At the species level, *A*. *omnicolens*, *F*. *nucleatum*, *P*. *lymphophilum*, and *B*. *longum* were differentially abundant in the “yes” group [Figure 6E]. In PROM comparisons, the “no” group was associated with adenosine biosynthesis and pyruvate fermentation, while the “yes” group was enriched in TCA cycle and glucose degradation pathways [Figure 6F].

## DISCUSSION

In this study, we characterized the microbiota of three maternal sources (oral, vaginal, and placental) and their potential transmission to the oral and gut microbiota of 18 healthy mother-infant dyads. This approach enabled us to explore the contribution of various maternal routes and perinatal factors to the initial colonization of the infant microbiome. Our findings support recent evidence of a distinct meconium microbiome and the absence of a placental microbiome. We also demonstrate significant shared bacterial taxa, particularly from the maternal to the infant oral cavity, and highlight the influence of perinatal factors on the microbial relationship between mother and infant.

One key question in infant microbiome development is whether the intrauterine environment is sterile and when initial microbial colonization occurs - during pregnancy or after birth. Recent studies suggest that fetal meconium lacks a detectable microbiota before birth^[40]^, though the presence of a microbiome in neonatal meconium remains debated^[6]^. The existence of a placental microbiome is similarly disputed^[41,42]^, and such samples are often categorized as “potential no (zero) biomass samples”^[6]^. To ensure accurate detection and minimize contamination, we included negative DNA extraction and sequencing controls and followed stringent protocols recommended for low biomass samples^[43,44]^. In our cohort, the core meconium microbiome consisted of genera such as *Staphylococcus*, *Bifidobacterium*, *Streptococcus*, *Enterococcus*, *Escherichia-Shigella*, *Delftia*, *Afipia*, *Cutibacterium*, and *Rothia*. Notably, *S. epidermidis*, common in colostrum and breast milk, was present in all meconium samples and has been previously identified in breastfed neonates’ meconium^[45-47]^. These findings align with studies suggesting that neonatal meconium reflects microbial communities acquired during and post-birth^[3,48-50]^. Functional analysis using PICRUSt2 revealed that the pentose phosphate pathway was the most abundant metabolic pathway in meconium. This pathway is crucial for generating NADPH, which is needed for biosynthetic reactions, and ribose-5-phosphate for nucleotide synthesis. Other prevalent pathways included L-isoleucine biosynthesis, branched-chain, aromatic amino acid biosynthesis, and glycolysis, indicating roles in energy production and biosynthesis of essential molecules. Regarding placental microbiota, we did not detect any non-contaminant ASVs, aligning with other studies that report the absence of a placental microbiome^[51-53]^. Exploratory analysis without decontamination steps showed that ASVs in placental samples matched those in negative controls, with phyla (Actinobacteriota, Firmicutes, Proteobacteria), families (*Bacillaceae*, *Corynebacteriaceae*, *Micrococcaceae*, *Streptococcaceae*, *Xanthobacteraceae*), and genera (*Afipia*, *Bacillus*, *Corynebacterium*, *Enhydrobacter*, *Micrococcus*, *Streptococcus*) similar in presence and relative abundance. These taxa are known contaminants from the laboratory^[54,55]^. Thus, our findings support the consensus that microbial colonization typically occurs at birth and that replicating microbes are absent in healthy pregnancies without clinical infections^[42]^.

The second aim of our study was to investigate the contribution of maternal microbial sources (vagina, oral cavity, and placenta) to their infants’ oral and gut microbiomes. Focusing first on the infant oral microbiome, its composition was consistent with previous studies, dominated by *Streptococcus*, *Rothia*, *Prevotella*, *Neisseria*, *Escherichia-Shigella*, *Gemella*, and *Haemophilus*^[56]^. Early colonizers like *S. salivarius*, *S. oralis*, *R. mucilaginosa*, *S. epidermidis*, and *F. nucleatum* were abundant^[57-59]^. Maternal oral samples, predominantly containing *Prevotella*, *Streptococcus*, *Veillonella*, *Rothia*, *Neisseria*, and *Haemophilus*, were similar to findings in other cohorts^[60-62]^. Species like *R. mucilaginosa*, *H. parainfluenzae*, *P. melaninogenica*, and *F. nucleatum* were prevalent^[63-65]^. The vaginal microbiome demonstrated a unique composition compared to meconium and oral microbiomes, with significantly lower diversity^[18]^. Dominant genera included *Peptoniphilus*, *Lactobacillus*, *Finegoldia*, *Corynebacterium*, and *Anaerococcus*, indicating community state type 4-A and 4-B with lower lactic acid bacteria and higher anaerobic bacteria^[62,66]^. Species such as *F. magna* and *P. faecalis*, both associated with bacterial vaginosis, were found in most samples^[67,68]^. The metabolic pathways identified were consistent with those reported across vaginal samples^[69]^. Vertical transmission of microbiota is primarily influenced by the maternal gut, but our study focused on the maternal oral and vaginal contributions to the infant’s oral and gut microbiomes^[70]^. Overall, infant and maternal oral microbiomes were similar, except for *Escherichia-Shigella*, *Cutibacterium*, and *Corynebacterium* detected in infants. On average, 45 ASVs were shared between mother-infant pairs, accounting for 65% of reads in infant samples. While perinatal factors did not significantly affect sharing, Kageyama *et al*. found greater acquisition of maternal oral bacteria in formula-fed infants^[71]^. Shared taxa included *Streptococcus*, *Veillonella*, *Neisseria*, *Haemophilus*, and *Fusobacterium*, consistent with previous studies^[63]^. *S. oralis* was found in all oral samples and is known as a primary colonizer in both infants and adults^[72,73]^. Other highly shared species like *H. parainfluenzae* and *R. mucilaginosa* are also commensal microbiota, while *F. nucleatum*, associated with periodontal disease, was present in most dyads^[74,75]^. These findings align with reports that around 70% of the neonatal oral microbiota is maternally derived^[76,77]^. Regarding vaginal-to-oral transmission, approximately 15 bacterial taxa, accounting for 15% of reads in infant samples, were shared. NB resulted in significantly higher sharing between maternal vaginal and infant oral microbiomes compared to CS (Mann Whitney, *P* = 0.045), similar to other studies^[19,78]^. *C. pyruviciproducens*, present in all dyads, was the only shared species between oral and vaginal samples. It is a pyruvic acid producer and potential pathogen^[79]^. *F. nucleatum* was also highly prevalent, present in 15 dyads. For maternal oral transfer to meconium, an average of eight bacterial taxa (6% of reads) were shared, with *R. mucilaginosa* being the only universally shared species, known for its anti-inflammatory properties^[80]^. Vaginal-to-meconium sharing included 38 taxa, accounting for 21% of reads, with *B. longum* found in all dyads. *B. longum* is a key commensal of the gut microbiota, with beneficial effects on neonatal health^[81-83]^.

The final aim of this study was to explore factors that influence microbial strain inheritance and/or selection in infants. These factors provide insight into the development of early-life medical conditions and could inform new preventive treatments. Maternal antibiotic use had a significant impact on the beta diversity of meconium. Opportunistic pathogens like *Gemella* and *Fusobacterium* spp. were differentially abundant in infants of mothers who received antibiotics, with an increase in heme biosynthesis pathways, critical for bacterial virulence^[84,85]^. Similar results were found in maternal saliva, where *Prevotella* and *Fusobacterium* spp., linked to periodontitis, were abundant in antibiotic-exposed groups^[86]^. Vaginal microbiome diversity also increased with antibiotic use, reducing *Lactobacillus* spp., known for maintaining vaginal health^[87]^. Functional pathways, including those related to succinate production, which supports bacterial vaginosis, were enriched in the antibiotic group^[88-90]^. Delivery mode affected meconium diversity, with *Intestinibacter* and *Veillonella* spp. prominent in CS infants^[91]^. NB infants displayed more commensal bacteria like *Bifidobacterium*, linked to lactic acid production and gut health^[92]^. In the infant oral microbiome, delivery mode shaped the prevalence of *Bifidobacterium longum* and *Veillonella parvula* in NB infants, whereas *L. mirabilis*, associated with periodontal health, was enriched in CS infants^[93,94]^. CS delivery also affected maternal oral microbiota, enriching *Fusobacterium* spp., associated with periodontitis, while NB mothers had higher levels of *Streptococcus oralis* and *Actinomyces*^[95,96]^. Feeding type played a key role in microbial colonization, with breastfed infants enriched in *Bacteroides* spp., capable of metabolizing human milk oligosaccharides (HMOs)^[97,98]^. Formula-fed infants had a more diverse microbiota, including *Blautia* spp. and *Dorea* spp., typically seen in more mature gut microbiomes^[99]^. Breastfeeding was linked to health-promoting metabolic pathways, including vitamin K2 and vitamin B6 biosynthesis, essential for immune function and metabolism^[100]^. Gender differences were limited, with no significant functional shifts in the meconium microbiome, though certain taxa, such as *B. animalis* and *B. uniformis*, were more abundant in females^[101]^. These gender-specific differences remain poorly understood but are likely influenced by hormonal and immune interactions^[102]^. PROM impacted meconium, maternal oral, and vaginal microbiomes. PROM increased alpha diversity in the vaginal microbiome and reduced *Lactobacillus* spp., a critical protector of vaginal health^[103]^. In maternal oral samples, the pathogen *Capnocytophaga leadbetteri* was enriched, but PROM was also associated with pathogen-clearing pathways, such as nitrate reduction^[104]^. These results align with studies suggesting a lack of *Lactobacillus* spp. is linked to PROM occurrence, and clinical trials have explored probiotic treatments for PROM^[105]^.

There are several limitations to this study. Firstly, the use of a small subset of samples may have limited the ability to draw precise and broadly applicable conclusions regarding the impact of perinatal factors on the infant microbiome. A larger sample size would provide greater statistical power and more robust analyses. Additionally, the cross-sectional design prevents tracking microbiome changes over time, highlighting the need for longitudinal studies to understand its development and environmental influences. The limitations of 16S rRNA gene sequencing also restrict the ability to reach definitive conclusions, as it provides lower taxonomic resolution than shotgun sequencing, often limiting identification to the genus level. This hinders differentiation between pathogenic and non-pathogenic strains within genera, making it challenging to infer vertical transmission with certainty. However, genus-level identification may still provide valuable insights, particularly in cases where unusual or rare genera are observed, which could suggest a potential link to maternal sources^[106]^. The methodology may also artificially inflate bacterial species abundance due to early PCR kinetics. Future studies could benefit from targeted qPCR, amplification-free metagenomics, FISH, SEM, microbial culturing, and control DNA spiking to enhance absolute abundance calculation. The use of PICRUSt2 to predict metabolic function also has limitations. Its predictions are biased toward established reference genomes, reducing the identification of rare, environment-specific functions, although the expansion of genome databases is mitigating this bias. Moreover, the amplicon-based nature of PICRUSt2 limits its ability to discern strain-specific functionality; shotgun metagenomics would overcome this, allowing for accurate gene abundance measurement and strain-level differentiation. Finally, there is a need to investigate how multiple perinatal factors interact in shaping the infant microbiome. For example, breastfeeding can modify colonization patterns in C-section-born infants^[107]^. Understanding transmission routes and the influence of perinatal factors on the mother-infant microbial bond is crucial for developing therapeutic interventions and next-generation probiotics to support infant health. Finally, it is important to clarify that while the CS rate in this subset (*n* = 18) was 44%, the rate across the full recruited cohort (*n* = 63) was 36.6%, which aligns closely with the national average in Ireland and in the recruitment hospital during the study period (~35%)^[108]^ and with more recent figures (~39%)^[109]^.

In conclusion, this study highlights the significant role of maternal sources, particularly the oral and vaginal microbiomes, in microbial sharing with infants. We observed that, on average, 45 ASVs were shared between mother-infant oral samples, accounting for 65% of reads in infant oral microbiota. Approximately 15 vaginal bacterial taxa (15% of infant reads) were shared, with significantly higher transmission following vaginal delivery (Mann–Whitney *P* = 0.045). Our findings confirm the presence of a distinct meconium microbiome - comprising taxa such as *S. epidermidis*, *Bifidobacterium*, and *Streptococcus* and support the absence of a measurable placental microbiome, consistent with negative control profiles.

Perinatal factors, including delivery mode, maternal antibiotic use, and feeding type, influenced microbial diversity and functional pathways. For instance, maternal antibiotic exposure was associated with increased abundance of *Gemella* and *Fusobacterium* and enrichment of heme biosynthesis pathways in meconium, while breastfeeding promoted pathways involved in vitamin K2 and B6 biosynthesis. Additionally, feeding type influenced microbial composition, with *Bacteroides* enriched in breastfed infants and *Blautia* and *Dorea* in formula-fed infants. These results underscore the complex and dynamic interactions shaping early microbial colonization, highlighting critical maternal contributions and the modulating role of perinatal exposures. Future longitudinal studies incorporating strain-resolved metagenomics are necessary to track microbial development and transmission patterns over time, with the ultimate goal of informing next-generation probiotic and therapeutic interventions to support infant health.

## DECLARATIONS

### Availability of data and materials

All 16S rRNA gene sequencing data have been deposited into the NCBI Sequence Read Archive, BioProject ID SUB14944107. Other data will be made available from the corresponding author upon reasonable request.

### Copyright

© The Author(s) 2025.
