# Supplementary material for: Perinatal factors influencing the earliest establishment of the infant microbiome
Source: Microbiome Res Rep. 2025 Jun 12;4(2):24. doi: 10.20517/mrr.2024.92 (PMC12369391; doi:10.20517/mrr.2024.92)
Supplement: Supplementary file 1 [file mrr-4-2-24-SupplementaryMaterials.pdf]

Supplementary Materials

Perinatal factors influencing the earliest establishment of the infant microbiome

Kevin Linehan[1,2,3], Kiera Healy[1,2], Eimear Hurley[4], Carol Anne O'Shea[4], C. Anthony Ryan[2,4], R. Paul Ross[2,3], Catherine Stanton[1,2,5], Eugene M. Dempsey[2,4]

[1]Teagasc Food Research Centre, Fermoy, Cork P61 C996, Ireland.
[2]APC Microbiome Ireland, Biosciences Institute, University College Cork, Cork T12 YT20, Ireland.
[3]School of Microbiology, University College Cork, Cork T12 YN60, Ireland.
[4]Department of Paediatrics & Child Health and INFANT Centre, University College Cork, Cork T12 YN60, Ireland.
[5]VISTAMILK RESEARCH Centre, Teagasc Moorepark, Cork P61 C996, Ireland.

Correspondence to: Prof. Eugene M. Dempsey, Department of Paediatrics & Child Health and INFANT Centre, University College Cork, 5th Floor, Cork University Maternity Hospital, Wilton, Cork T12 YN60, Ireland. E-mail: g.dempsey@ucc.ie

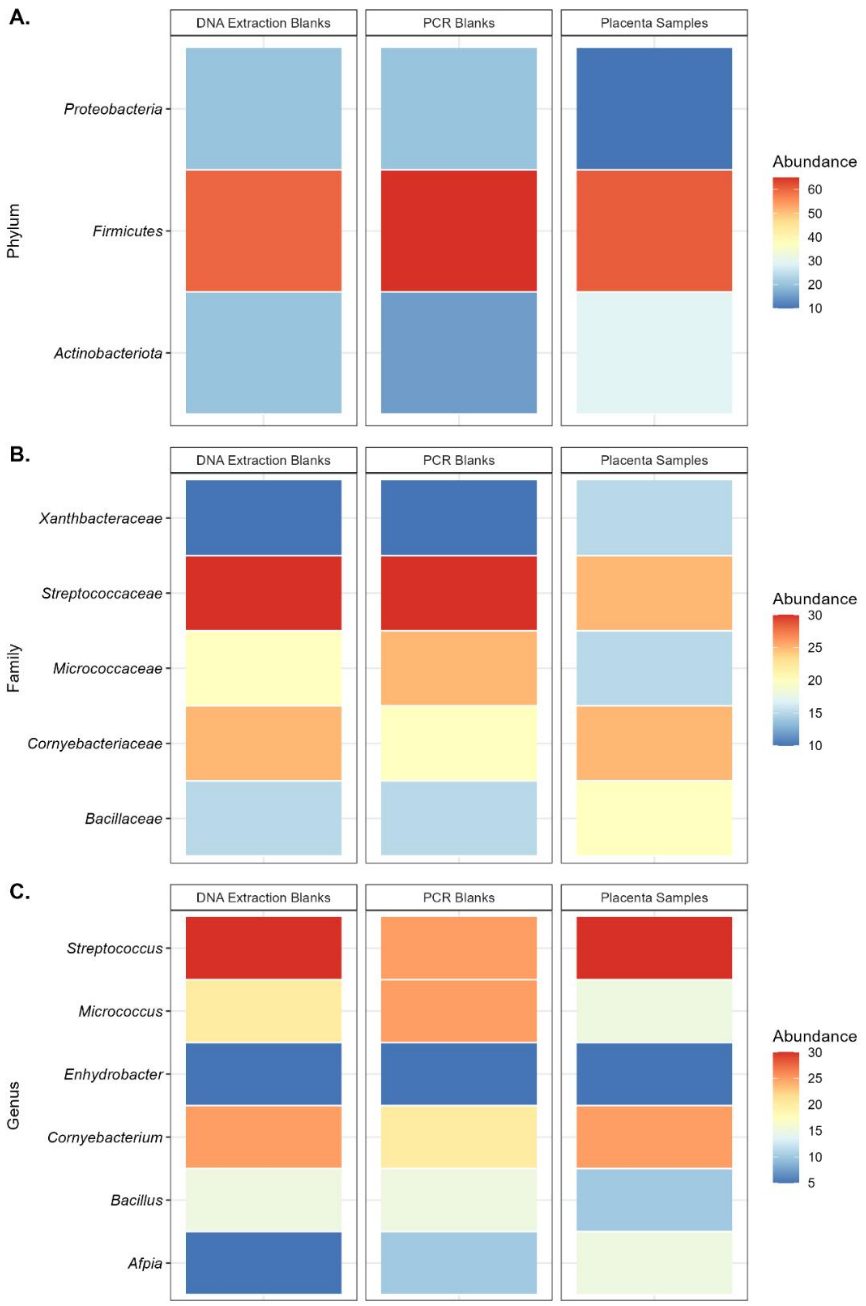

Supplementary Figure 1. Heat maps of average relative abundances at (A) phylum, (B) family, and (C) genus levels of DNA extraction blanks (n=5), amplicon PCR blanks (n=5), and placenta samples (n=8). The heat maps show the distribution of bacterial taxa across these sample types. Panel (A) displays the relative abundance of major phyla, with *Proteobacteria* and *Firmicutes* being predominant in placenta samples. Panel (B) illustrates the family-level composition, highlighting *Xanthobacteraceae* as more abundant in placenta samples compared to blanks. Panel (C) shows the genus-level relative abundance, with *Streptococcus* and *Micrococcus* being notably present in placenta samples.

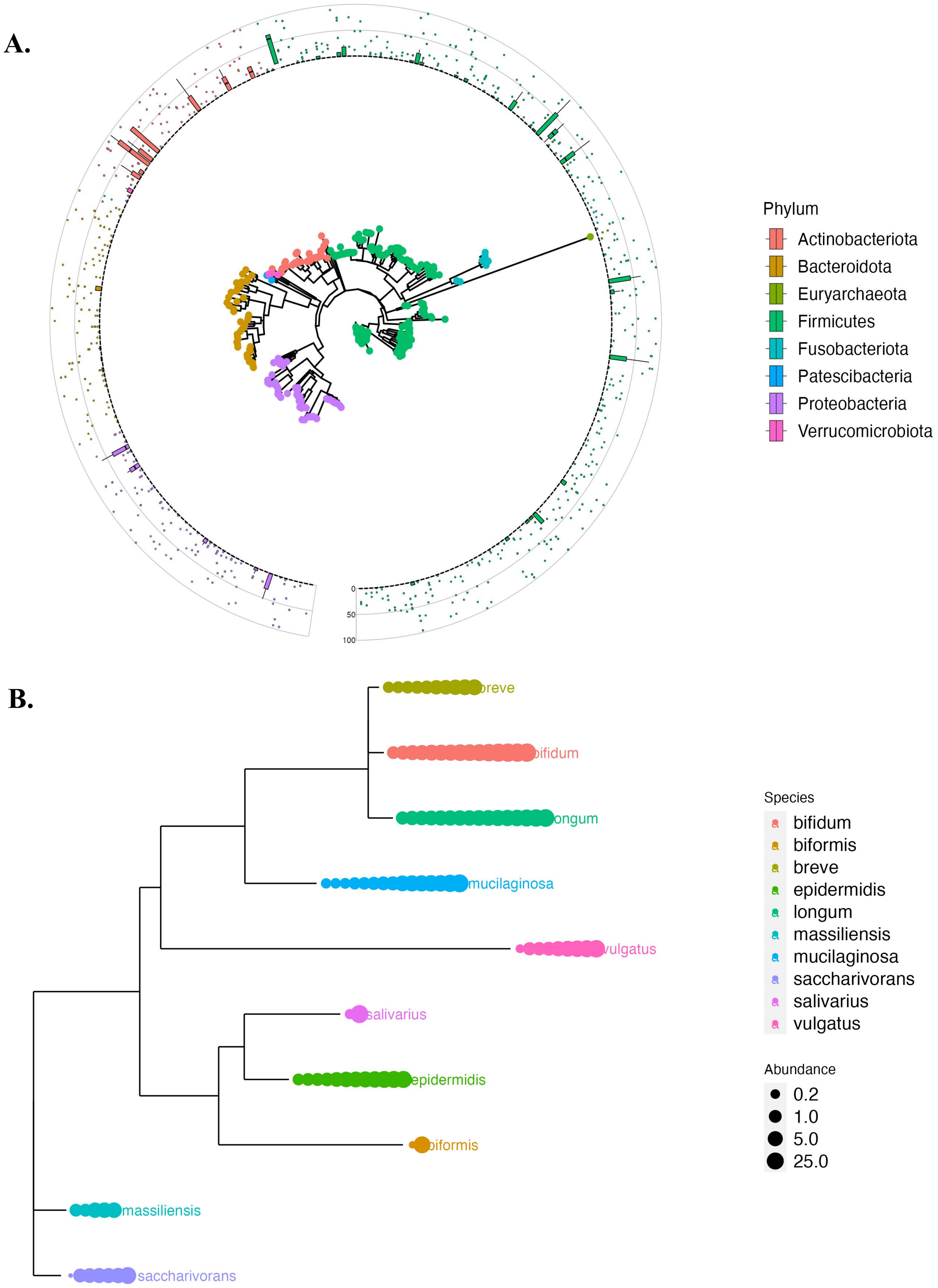
A.
0
50
100
Phylum
Actinobacteriota
Bacteroidota
Euryarchaeota
Firmicutes
Fusobacteriota
Patescibacteria
Proteobacteria
Verrucomicrobiota
B.
breve
bifidum
longum
mucilaginosa
vulgatus
salivarius
epidermidis
biformis
massiliensis
saccharivorans
Species
bifidum
biformis
breve
epidermidis
longum
massiliensis
mucilaginosa
saccharivorans
salivarius
vulgatus
Abundance
0.2
1.0
5.0
25.0

C.

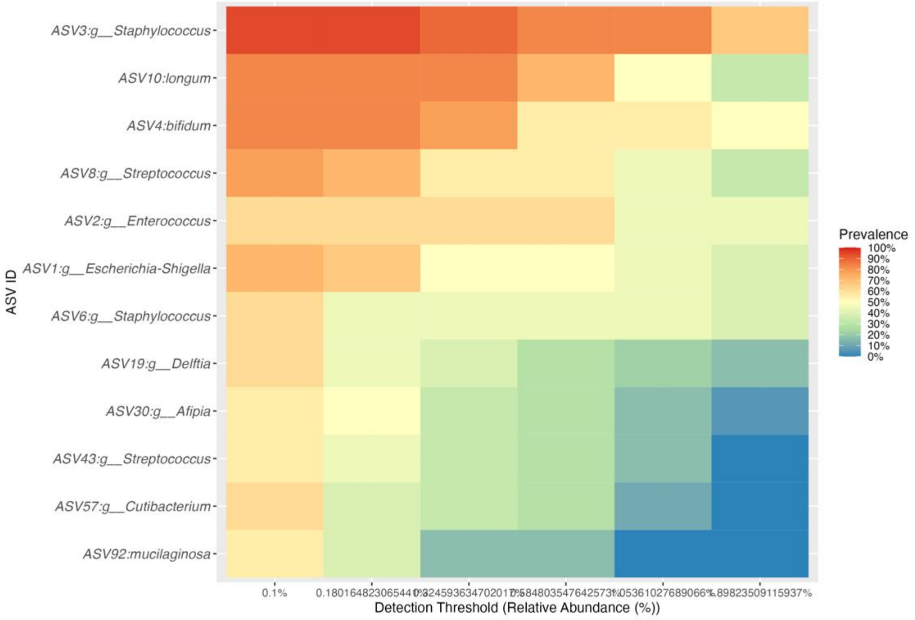

D.

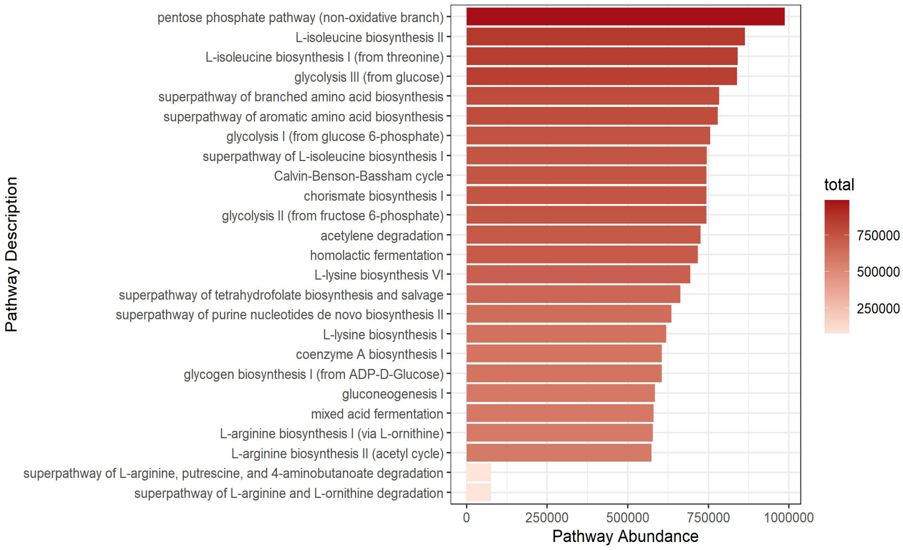

Supplementary Figure 2. (A) Phylogenetic tree with ASV relative abundance distribution shown as boxplots for Phylum-level abundances. Symbolic points coloured by phylum represent species abundances. (B) Phylogenetic tree with ASV relative abundance distribution for the top ten species in meconium samples. Node size represents relative abundance. (C) Heatmap of the core meconium microbiome, showing relative abundance and prevalence (> 0.001 cut-off and 50% cut-off, respectively) for the 18 samples analysed. ASVs are labelled with their ID and lowest taxonomic classification. (D) Predictive metabolic pathways associated with the meconium microbiome, displaying the top 25 abundant pathways based on abundance levels.

A.

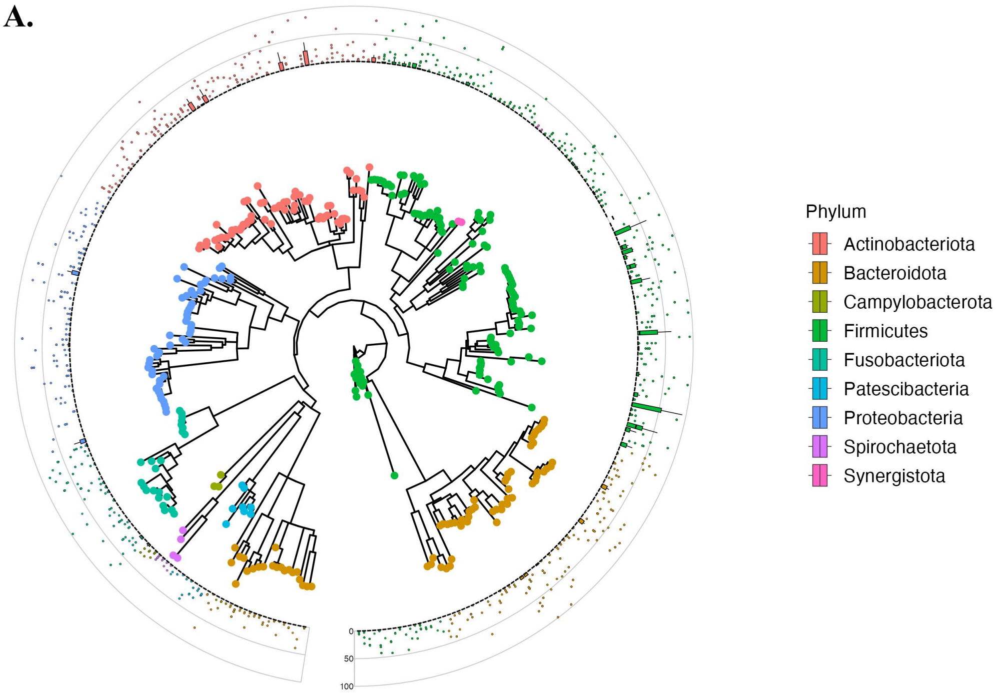
0
50
100
Phylum
Actinobacteriota
Bacteroidota
Campylobacterota
Firmicutes
Fusobacteriota
Patescibacteria
Proteobacteria
Spirochaetota
Synergistota

B.

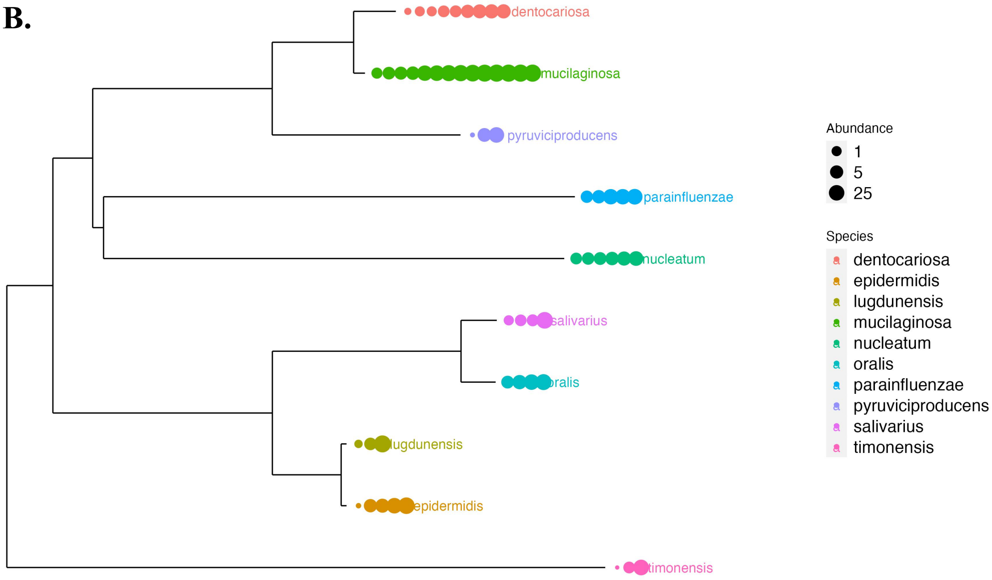
dentocariosa
mucilaginosa
pyruviciproducens
parainfluenzae
nucleatum
salivarius
oralis
lugdunensis
epidermidis
timonensis
Abundance
1
5
25
Species
dentocariosa
epidermidis
lugdunensis
mucilaginosa
nucleatum
oralis
parainfluenzae
pyruviciproducens
salivarius
timonensis

C.

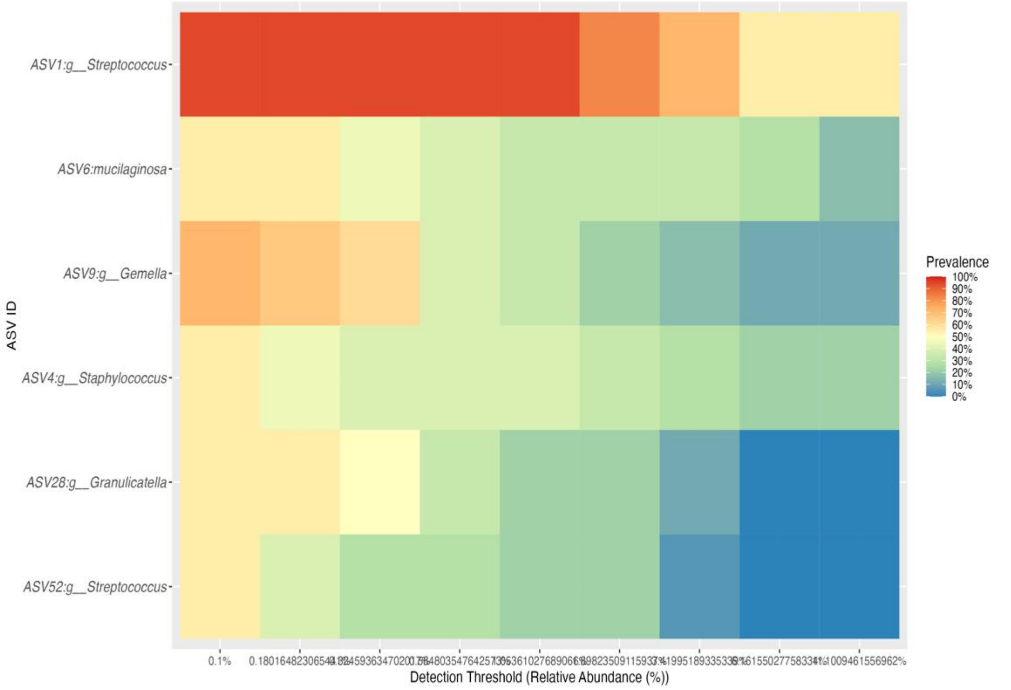

D.

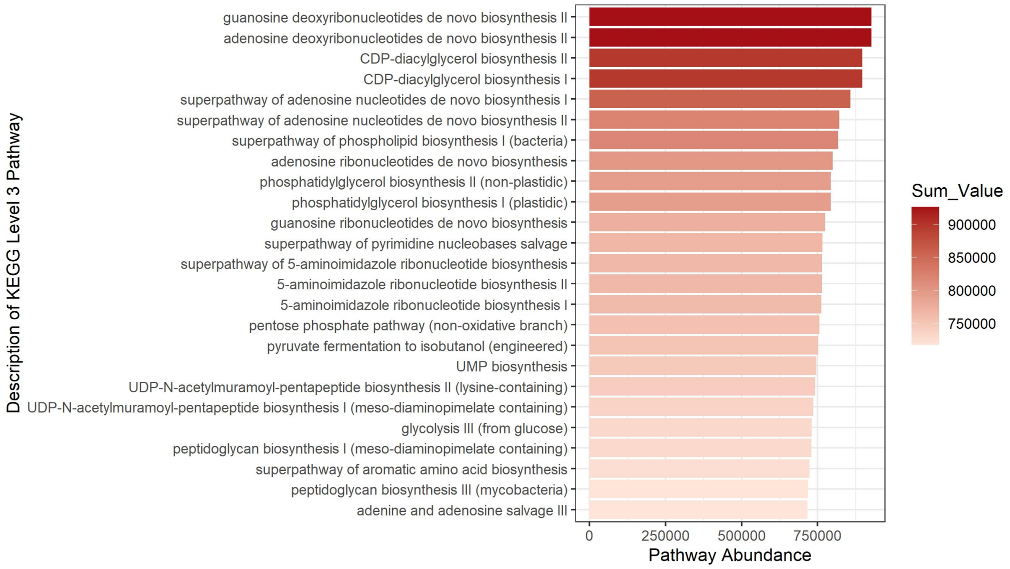

Supplementary Figure 3. (A) Phylogenetic tree with ASV relative abundance distribution shown as boxplots for Phylum-level abundances. Symbolic points coloured by phylum represent species abundances. (B) Phylogenetic tree with ASV relative abundance distribution for the top ten species in infant oral samples. Node size represents relative abundance. (C) Infant oral core microbiome heatmap, showing relative abundance and prevalence (> 0.001 cut-off and 50% cut-off, respectively) for the 18 samples analysed. ASVs are labelled with their ID and lowest taxonomic classification. (D) Predictive metabolic pathways associated with the infant oral microbiome, displaying the top 25 abundant pathways based on abundance level.

A.

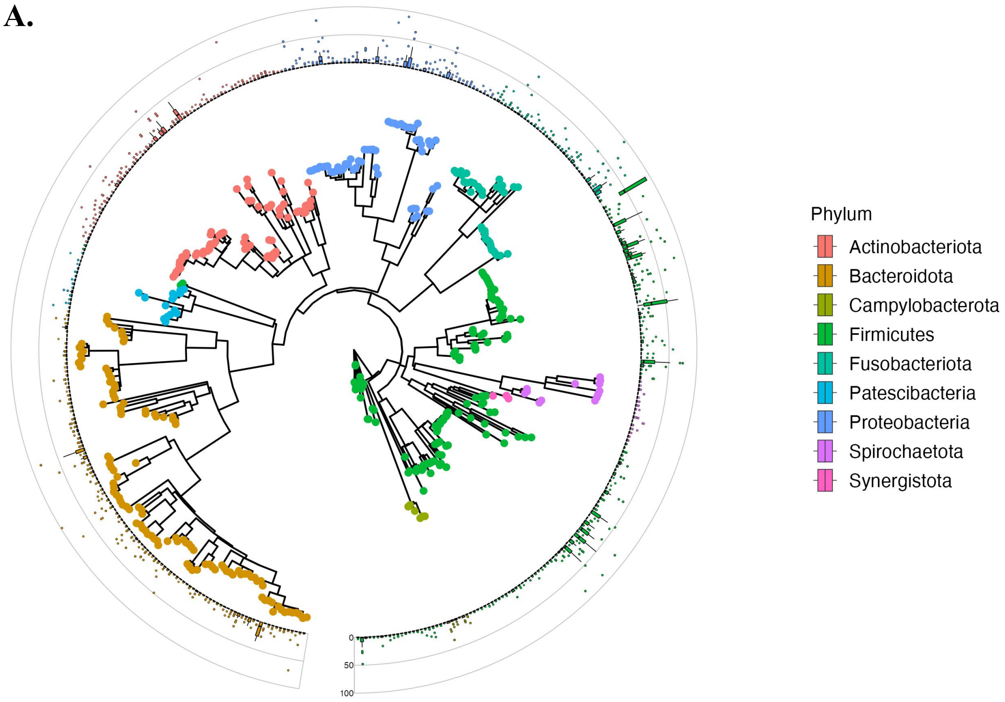
0
50
100
Phylum
Actinobacteriota
Bacteroidota
Campylobacterota
Firmicutes
Fusobacteriota
Patescibacteria
Proteobacteria
Spirochaetota
Synergistota

B.

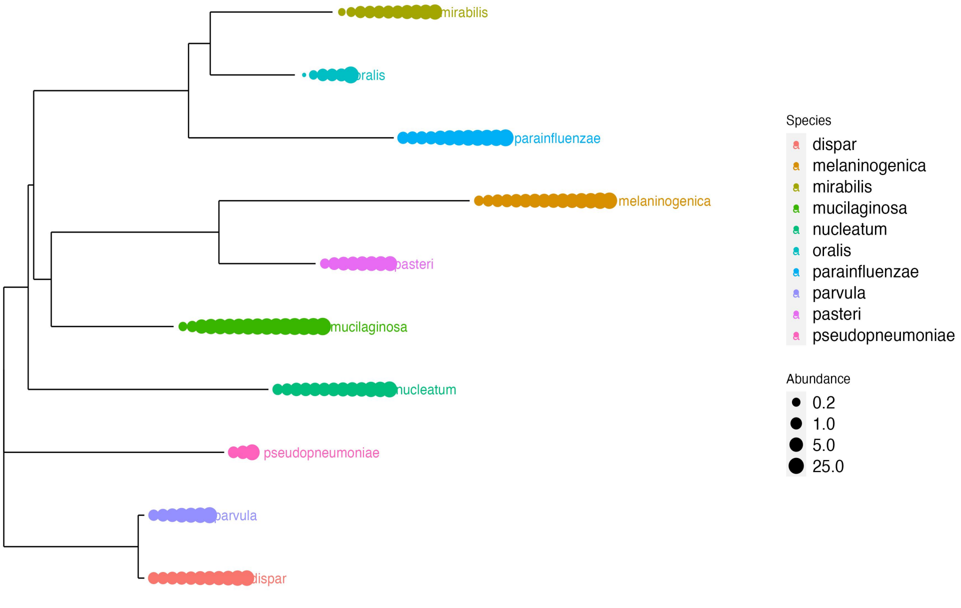
mirabilis
oralis
parainfluenzae
melaninogenica
pasteri
mucilaginosa
nucleatum
pseudopneumoniae
parvula
dispar
Species
dispar
melaninogenica
mirabilis
mucilaginosa
nucleatum
oralis
parainfluenzae
parvula
pasteri
pseudopneumoniae
Abundance
0.2
1.0
5.0
25.0

C.

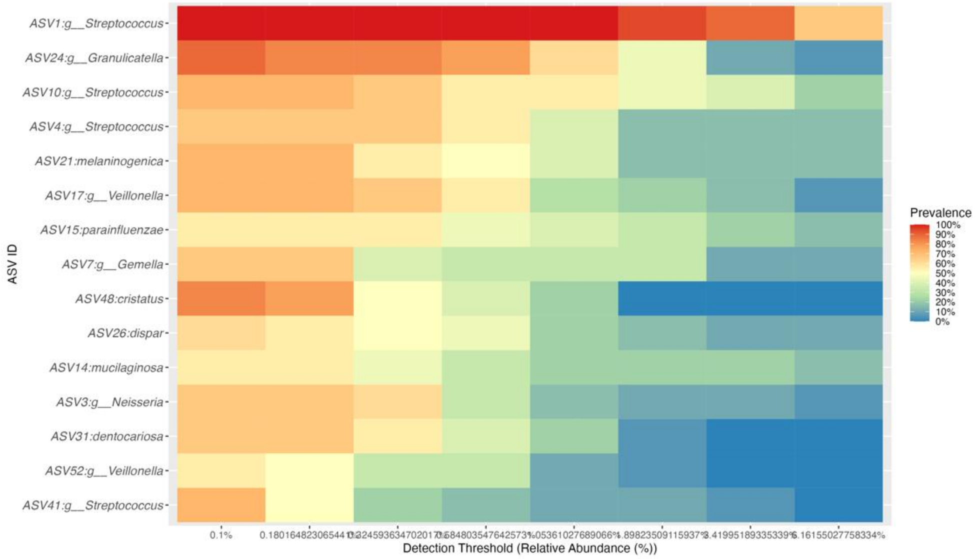

D.

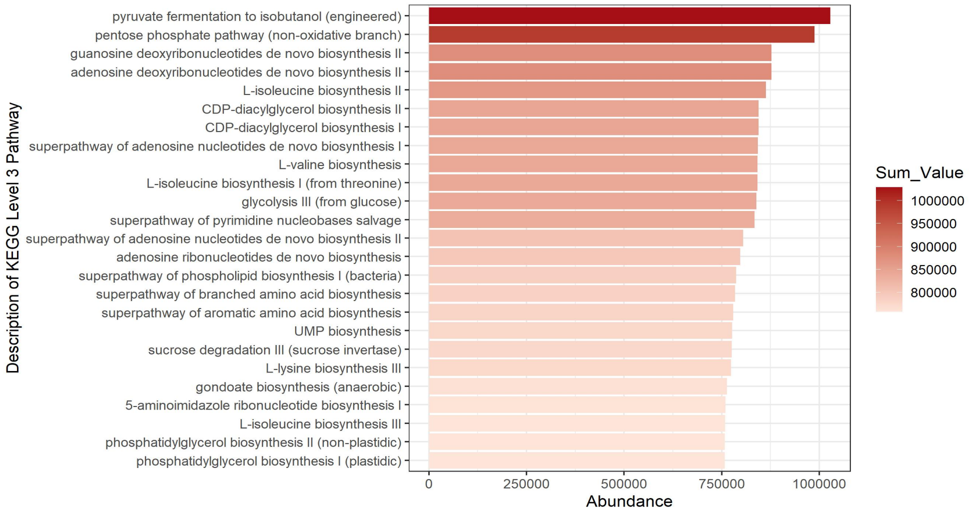

Supplementary Figure 4. (A) Phylogenetic tree with ASV relative abundance distribution shown as boxplots for phylum-level abundances. Symbolic points coloured by phylum represent species abundances. (B) Phylogenetic tree with ASV relative abundance distribution for the top ten species in maternal saliva samples. Node size represents relative abundance. (C) Maternal oral core microbiome heatmap, showing relative abundance and prevalence (> 0.001 cut-off and 50% cut-off, respectively) for the 18 samples analysed. ASVs are labelled with their ID and lowest taxonomic classification. (D) Predictive metabolic pathways associated with the maternal oral microbiome, displaying the top 25 abundant pathways based on abundance levels.

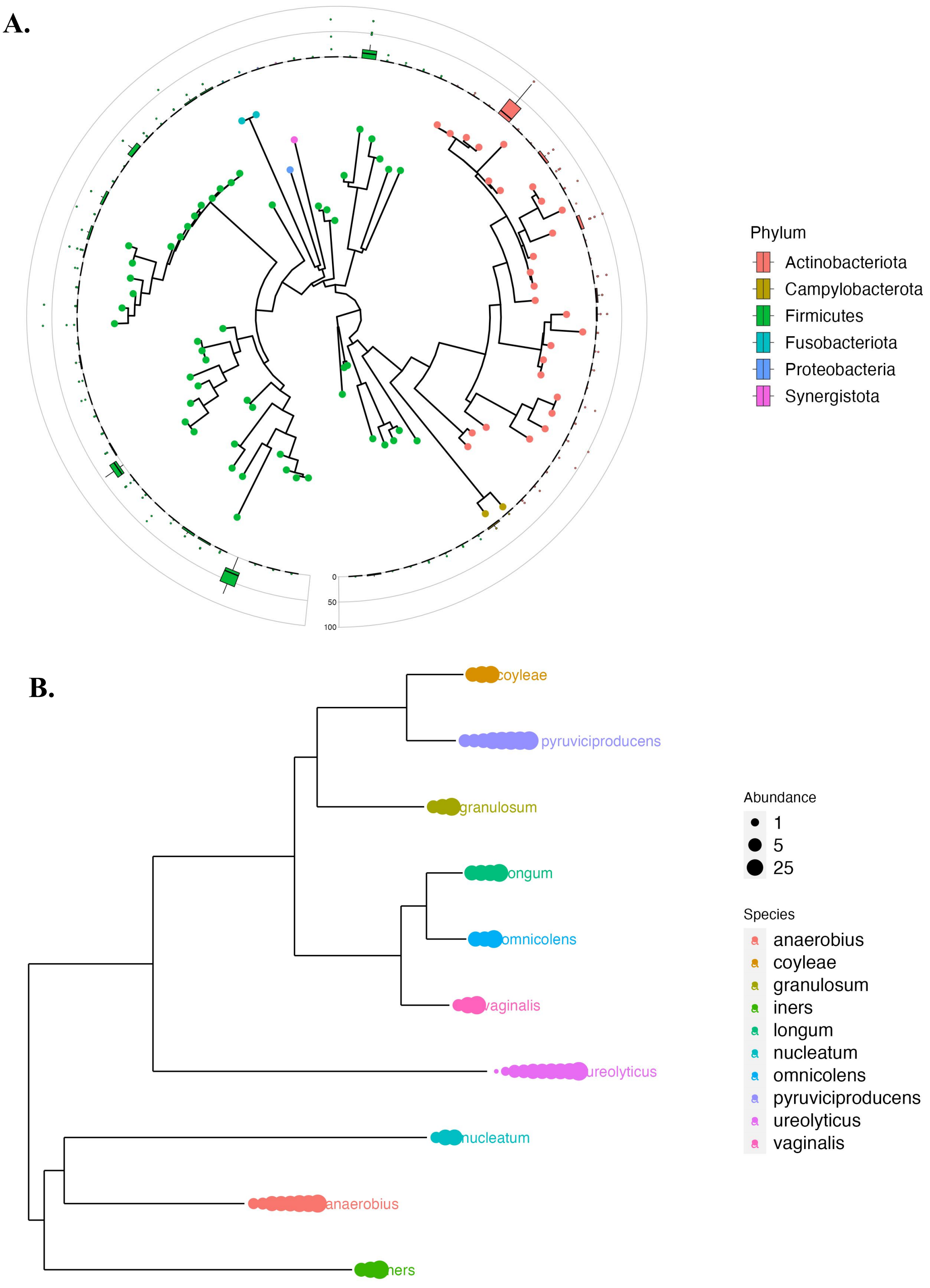
A.
0
50
100
Phylum
Actinobacteriota
Campylobacterota
Firmicutes
Fusobacteriota
Proteobacteria
Synergistota
B.
coyleae
pyruviciproducens
granulosum
longum
omnicolens
vaginalis
ureolyticus
nucleatum
anaerobius
iners
Abundance
1
5
25
Species
anaerobius
coyleae
granulosum
iners
longum
nucleatum
omnicolens
pyruviciproducens
ureolyticus
vaginalis

C.

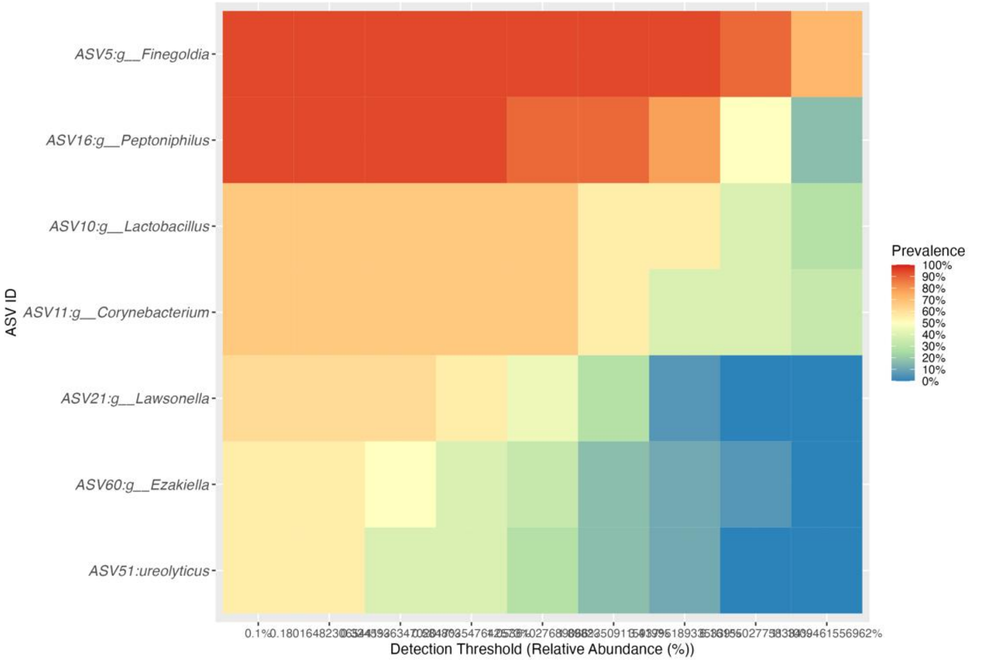

D.

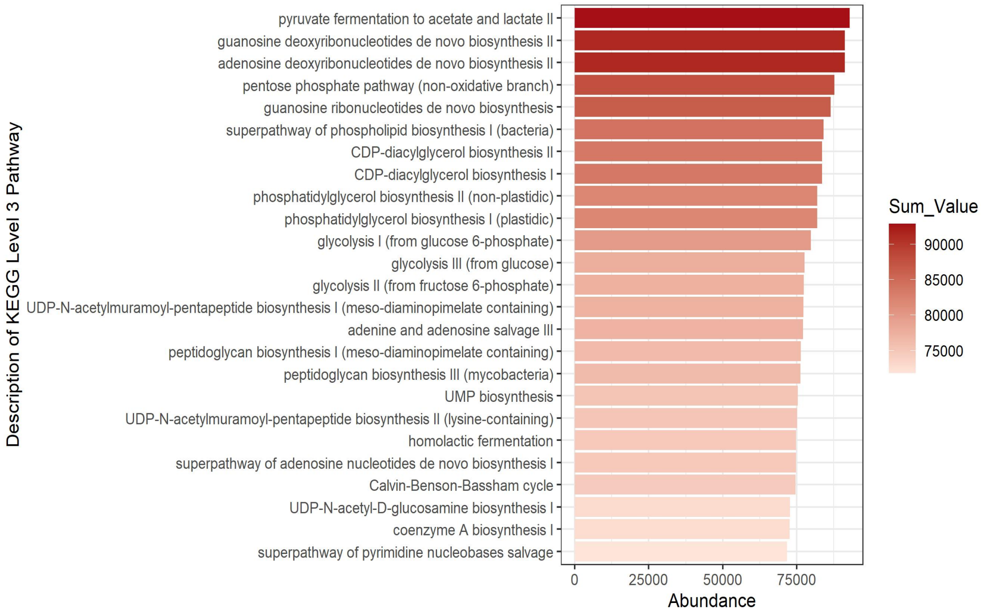

Supplementary Figure 5. (A) Phylogenetic tree with ASV relative abundance distribution shown as boxplots for phylum-level abundances. Symbolic points coloured by phylum represent species abundances. (B) Phylogenetic tree with ASV relative abundance distribution for the top ten species in vaginal samples. Node size represents relative abundance. (C) Vaginal core microbiome heatmap, showing relative abundance and prevalence (> 0.001 cut-off and 50% cut-off, respectively) for the 18 samples analysed. ASVs are labelled with their ID and lowest taxonomic classification. (D) Predictive metabolic pathways associated with the vaginal microbiome, displaying the top 25 abundant pathways based on abundance levels.

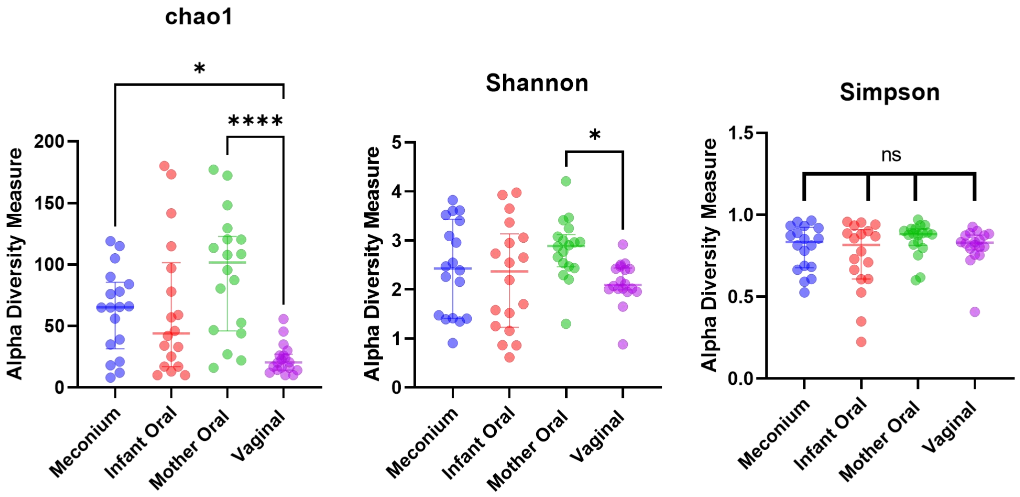

Supplementary Figure 6. Alpha diversity analysis of mother and infant samples. Statistical significance assessed by Mann-Whitney test ($p < 0.05$).

A.

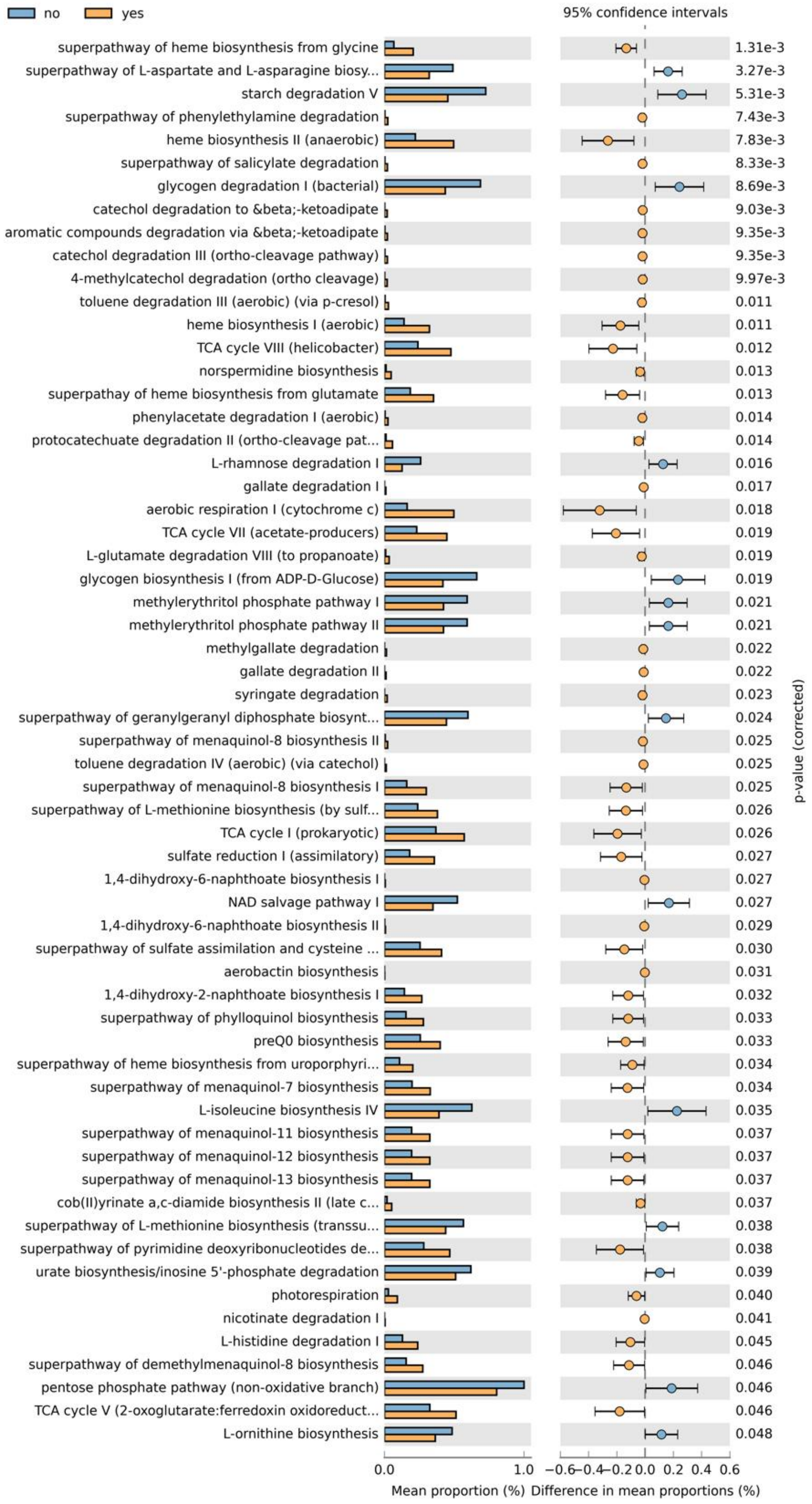
no
yes
95% confidence intervals
superpathway of heme biosynthesis from glycine
1.31e-3
superpathway of L-aspartate and L-asparagine biosy...
3.27e-3
starch degradation V
5.31e-3
superpathway of phenylethylamine degradation
7.43e-3
heme biosynthesis II (anaerobic)
7.83e-3
superpathway of salicylate degradation
8.33e-3
glycogen degradation I (bacterial)
8.69e-3
catechol degradation to β-ketoadipate
9.03e-3
aromatic compounds degradation via β-ketoadipate
9.35e-3
catechol degradation III (ortho-cleavage pathway)
9.35e-3
4-methylcatechol degradation (ortho cleavage)
9.97e-3
toluene degradation III (aerobic) (via p-cresol)
0.011
heme biosynthesis I (aerobic)
0.011
TCA cycle VIII (helicobacter)
0.012
norspermidine biosynthesis
0.013
superpathay of heme biosynthesis from glutamate
0.013
phenylacetate degradation I (aerobic)
0.014
protocatechuate degradation II (ortho-cleavage pat...
0.014
L-rhamnose degradation I
0.016
gallate degradation I
0.017
aerobic respiration I (cytochrome c)
0.018
TCA cycle VII (acetate-producers)
0.019
L-glutamate degradation VIII (to propanoate)
0.019
glycogen biosynthesis I (from ADP-D-Glucose)
0.019
methylerythritol phosphate pathway I
0.021
methylerythritol phosphate pathway II
0.021
methylgallate degradation
0.022
gallate degradation II
0.022
syringate degradation
0.023
superpathway of geranylgeranyl diphosphate biosynt...
0.024
superpathway of menaquinol-8 biosynthesis II
0.025
toluene degradation IV (aerobic) (via catechol)
0.025
superpathway of menaquinol-8 biosynthesis I
0.025
superpathway of L-methionine biosynthesis (by sulf...
0.026
TCA cycle I (prokaryotic)
0.026
sulfate reduction I (assimilatory)
0.027
1,4-dihydroxy-6-naphthoate biosynthesis I
0.027
NAD salvage pathway I
0.027
1,4-dihydroxy-6-naphthoate biosynthesis II
0.029
superpathway of sulfate assimilation and cysteine ...
0.030
aerobactin biosynthesis
0.031
1,4-dihydroxy-2-naphthoate biosynthesis I
0.032
superpathway of phylloquinol biosynthesis
0.033
preQ0 biosynthesis
0.033
superpathway of heme biosynthesis from uroporphyri...
0.034
superpathway of menaquinol-7 biosynthesis
0.034
L-isoleucine biosynthesis IV
0.035
superpathway of menaquinol-11 biosynthesis
0.037
superpathway of menaquinol-12 biosynthesis
0.037
superpathway of menaquinol-13 biosynthesis
0.037
cob(II)yrinate a,c-diamide biosynthesis II (late c...
0.037
superpathway of L-methionine biosynthesis (transsu...
0.038
superpathway of pyrimidine deoxyribonucleotides de...
0.038
urate biosynthesis/inosine 5'-phosphate degradation
0.039
photorespiration
0.040
nicotinate degradation I
0.041
L-histidine degradation I
0.045
superpathway of demethylmenaquinol-8 biosynthesis
0.046
pentose phosphate pathway (non-oxidative branch)
0.046
TCA cycle V (2-oxoglutarate:ferredoxin oxidoreduct...
0.046
L-ornithine biosynthesis
0.048
p-value (corrected)
0.0
1.0
Mean proportion (%)
−0.6
−0.4
−0.2
0.0
0.2
0.4
0.6
Difference in mean proportions (%)

B.

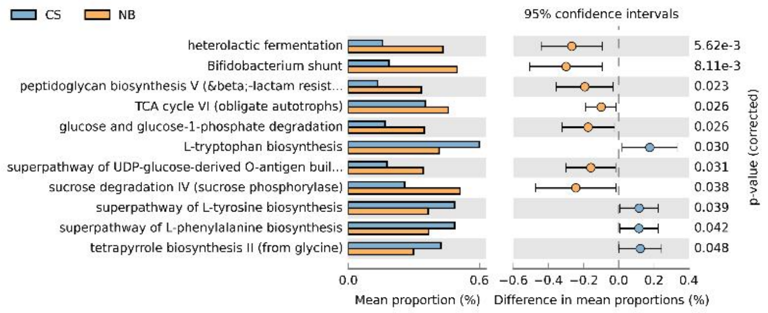

C.

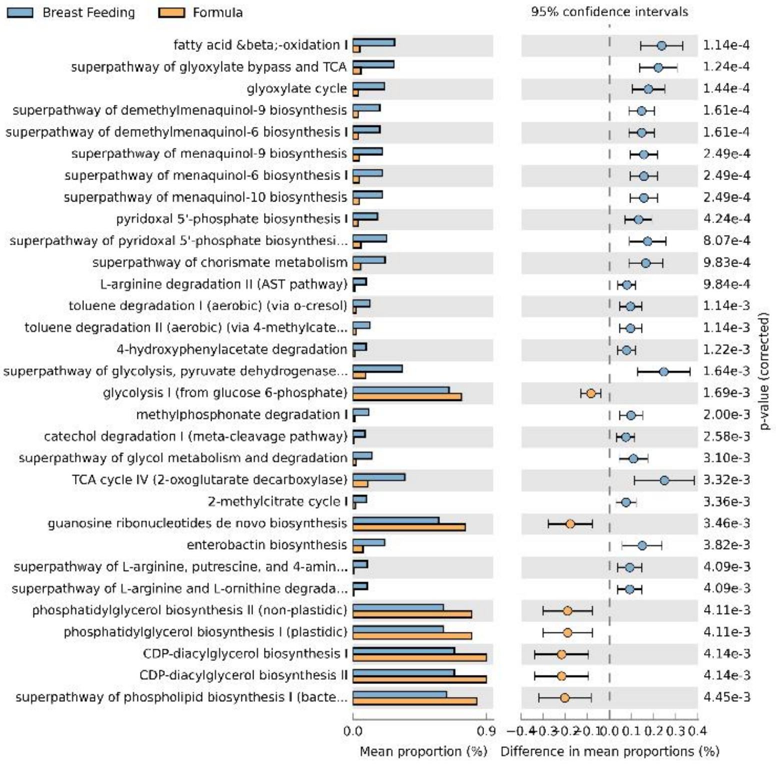

Supplementary Figure 7. Impact of perinatal factors on meconium microbiome functionality. Extended error bar plots comparing predicted KEGG functions using PICRUSt2. Bar plots show mean proportions of functional pathways, with error bars representing group differences. Error bars are color-coded based on the group with higher proportions. (A) Mother antibiotic usage. (B) Delivery Mode. (C) Feed Type. Welch's t-test was used with $p < 0.05$ (two-sided) as the significance threshold. Abbreviations: CS, C-section; NB, Natural Birth.

Supplementary Table 1. Forward primers and reverse primers used for 16S rRNA analysis

Primer	Sequence (5'–3')	Target Region	Expected Amplicon Size	Reference
Forward	TCGTCGGCAGCGTCAGATGTGTATAAGAGACAGCCTACGGGNGGCWGCAG	V3–V4	~550 bp	Illumina 16S Metagenomic Protocol
Reverse	GTCTCGTGGGCTCGGAGATGTGTATAAGAGACAGGACTACHVGGGTATCTAATCC	V3–V4	~550 bp	Illumina 16S Metagenomic Protocol
